# Functional Materials for Additive Manufacturing: Materials Design, Processing, and Emerging Applications

**DOI:** 10.3390/nano16140881

**Published:** 2026-07-17

**Authors:** Rashid Dallaev

**Affiliations:** Department of Physics, Faculty of Electrical Engineering and Communication, Brno University of Technology, Technická 2848/8, 616 00 Brno, Czech Republic; rashid.dallaev@vut.cz

**Keywords:** additive manufacturing, polymer nanocomposites, fused filament fabrication, nanoparticle reinforcement, graphene and carbon nanotubes, mechanical property enhancement, printability and rheology, anisotropy in 3D printing

## Abstract

Additive manufacturing (AM) has evolved from a rapid prototyping technique into a versatile platform for fabricating advanced functional materials and complex engineering components. While polymers remain the dominant material class due to their processability and tunable properties, recent developments have expanded AM to include high-performance composites, nanocomposites, and metallic materials. This review provides an overview of functional materials for additive manufacturing, emphasizing the relationships between material design, processing conditions, microstructure evolution, and resulting properties. Key functional polymer systems are discussed, including conductive, stimuli-responsive, elastomeric, high-performance, bio-based, and nanocomposite materials reinforced with nanoparticles, carbon nanomaterials, MXenes, and fibers. This review also examines processing–structure–property relationships common to polymer- and metal-based AM, highlighting the roles of anisotropy, defect formation, residual stresses, and post-processing in determining component performance. Finally, current challenges and emerging trends—including multi-material and 4D printing, machine learning-assisted optimization, and digital materials design—are discussed. Overall, the review highlights how advances in materials science and intelligent manufacturing are expanding the capabilities of additive manufacturing for multifunctional engineering and biomedical applications.

## 1. Introduction

**Figure 1:**
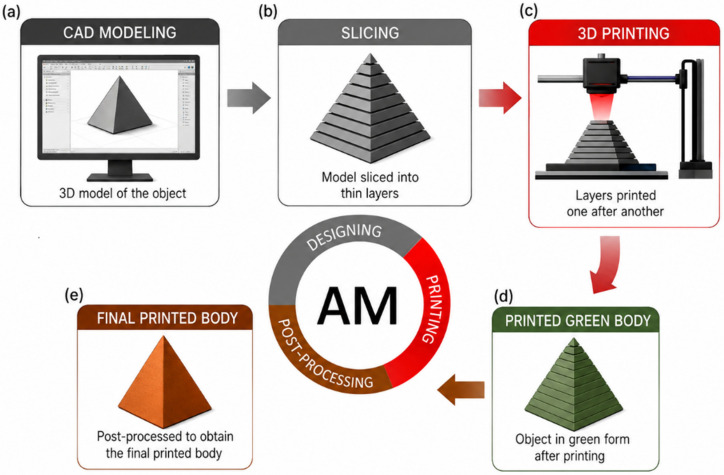
Illustration of the AM fabrication route. A 3D object is first designed and digitally sliced into individual layers (**a**,**b**). The object is then fabricated through layer-by-layer deposition (**c**), yielding a green body (**d**) that undergoes post-processing to generate the final product (**e**). (Reproduced from [3]; this figure is available under Open Access).

Additive manufacturing (AM), commonly referred to as three-dimensional (3D) printing, has emerged as a transformative manufacturing paradigm that enables the fabrication of complex structures through the layer-by-layer deposition of materials guided by digital models [1,2,3]. Unlike conventional subtractive or formative manufacturing techniques, AM offers unprecedented design freedom, allowing the production of geometrically intricate architectures that are otherwise difficult or impossible to achieve using traditional processes [4,5]. Over the past decade, rapid advancements in AM technologies—including material extrusion, powder bed fusion, stereolithography, and direct ink writing—have significantly expanded its industrial relevance, enabling applications across aerospace, biomedical engineering, energy systems, and electronics [6,7,8]. Recent reviews emphasize that in recent years, the field has witnessed accelerated progress not only in processing technologies but also in material systems, particularly polymer-based materials, which remain the most widely used class in AM due to their versatility, processability, and tunable properties [9,10,11,12]. Figure 1 provides a schematic illustration of the layer-wise fabrication process that transforms a digital design into a physical object through a sequence of interconnected steps. The workflow begins with computer-aided design (CAD) and model slicing, followed by the deposition of successive layers to build the desired geometry. The resulting green body is subsequently subjected to post-processing operations to achieve the final properties and dimensions of the printed component.

Polymers have played a central role in the evolution of AM, initially serving as model materials for rapid prototyping and visualization [13,14]. Early polymer-based AM systems primarily focused on producing structural components with adequate mechanical strength and dimensional accuracy. However, the scope of polymer AM has significantly expanded in recent years, transitioning from purely structural applications toward the development of functional materials capable of exhibiting advanced properties such as electrical conductivity, thermal management, shape memory, self-healing, and stimuli responsiveness [15]. This shift reflects a broader paradigm change in which AM is increasingly viewed not only as a fabrication tool but also as an enabling platform for designing materials with integrated functionalities at multiple length scales [16].

The evolution from structural to functional polymers in AM is driven by the growing demand for multifunctional devices and systems in emerging technological domains. For instance, functional polymer composites incorporating conductive fillers, nanoparticles, or continuous fibers have been developed to enhance electrical, thermal, and mechanical performance simultaneously [17,18]. Similarly, smart polymers such as shape memory polymers and self-healing materials have enabled the realization of 4D printing concepts, where printed structures can respond dynamically to external stimuli such as temperature, light, or moisture [19,20]. These advancements have opened new opportunities for applications in soft robotics, wearable electronics, biomedical implants, and energy storage devices, highlighting the critical role of functional polymers in advancing the capabilities of AM technologies [21].

Despite these advancements, the transition toward functional polymer systems in AM presents several challenges related to material design, processing compatibility, and performance optimization. The intrinsic properties of polymers, such as viscosity, thermal behavior, and curing kinetics, must be carefully tailored to meet the requirements of specific AM processes while maintaining the desired functional performance [22,23]. Furthermore, issues such as interlayer adhesion, anisotropy, and defects introduced during layer-by-layer fabrication can significantly affect the mechanical and functional properties of printed components [24]. Addressing these challenges requires a comprehensive understanding of the interplay between material formulation, processing parameters, and structure–property relationships, underscoring the importance of materials innovation in the field of AM [25,26].

Materials innovation has therefore become a central focus in the development of next-generation AM technologies. Recent research has emphasized the design of advanced polymer systems, including high-performance thermoplastics, photopolymer resins, bio-based polymers, and hybrid nanocomposites, to overcome the limitations of conventional materials [27,28,29]. In particular, the integration of nanomaterials and functional additives has enabled the tailoring of polymer properties at the molecular and nanoscale levels, leading to enhanced functionality and performance in printed structures [15]. Additionally, the emergence of multi-material additive manufacturing has provided new opportunities to fabricate heterogeneous structures with spatially varying properties, further expanding the design space for functional applications [30]. These developments highlight the critical role of materials design in unlocking the full potential of AM for advanced applications.

Another important trend in the field is the increasing emphasis on sustainability and circular economy principles in polymer-based AM. The use of bio-derived polymers, recyclable materials, and waste-derived feedstocks has gained significant attention as researchers seek to reduce the environmental impact of AM processes [31]. At the same time, efforts are being made to improve the recyclability and lifecycle performance of printed polymer components, particularly in powder-based processes where material reuse is a key consideration [32]. These initiatives reflect the growing recognition that sustainable materials development is essential for the long-term viability and scalability of AM technologies.

In parallel with materials advancements, significant progress has been made in computational design, simulation, and process optimization for polymer AM. Advanced modeling approaches and data-driven techniques are increasingly being used to predict material behavior, optimize printing parameters, and design complex architectures with tailored properties [33,34,35]. The integration of artificial intelligence and machine learning into the AM workflow has further accelerated the discovery and development of new materials, enabling more efficient exploration of the vast design space associated with functional polymers [36]. These developments are expected to play a crucial role in bridging the gap between laboratory-scale innovations and industrial-scale applications.

Given the rapid progress and increasing complexity of the field, there is a growing need for comprehensive reviews that provide a systematic overview of functional polymers for AM, covering aspects ranging from materials design and processing strategies to applications and future perspectives [37,38]. While several recent studies have addressed specific aspects of polymer AM, such as process optimization, composite materials, and individual applications, a holistic understanding of functional polymer systems and their integration into AM processes remains limited [9,39]. In particular, the relationships between material structure, processing conditions, and functional performance are not yet fully understood, highlighting the need for further research and synthesis of existing knowledge [13,22].

In this context, the present review aims to provide a comprehensive overview of functional polymers for additive manufacturing, with a focus on the design, development, and application of advanced polymer systems. The scope of this review encompasses (i) the fundamental principles of polymer-based AM processes, (ii) the design and synthesis of functional polymers and composites, (iii) the integration of multifunctional properties into printed structures, and (iv) emerging applications across various fields, including electronics, energy, healthcare, and smart systems. By critically analyzing recent literature published since 2022, this review seeks to identify key trends, challenges, and opportunities in the field, thereby providing valuable insights for researchers and practitioners working at the intersection of materials science and additive manufacturing.

Overall, the convergence of advanced materials design and additive manufacturing technologies is driving a new era of innovation in polymer science. The transition from structural to functional polymers represents a fundamental shift in the role of AM, transforming it from a prototyping tool into a powerful platform for fabricating next-generation devices and systems with tailored functionalities [13,31,40]. Continued advancements in materials development, process optimization, and application-driven research are expected to further expand the capabilities of functional polymer AM, paving the way for new technological breakthroughs in the years to come [27,41,42].

## 2. Process–Structure–Property Relationships in Polymer Design for Additive Manufacturing

Additive manufacturing (AM) has become a major route for producing polymer parts because it enables layer-wise fabrication of complex geometries, low-volume customization, and design freedom that are difficult to achieve by conventional molding or machining [37,43]. Polymers are especially attractive AM feedstocks because they can be engineered to combine low density and corrosion resistance with tailored mechanical, thermal, electrical, fire-resistant, optical, and biocompatible properties [10,44]. In practice, however, AM is not a single materials problem: each process class imposes different chemical, physical, and engineering requirements on the polymer feedstock, and material design must be matched to the printing route rather than chosen in a process-agnostic way [13,45,46].

### 2.1. Process-Specific Feedstock Design in Polymer AM

**Figure 2:**
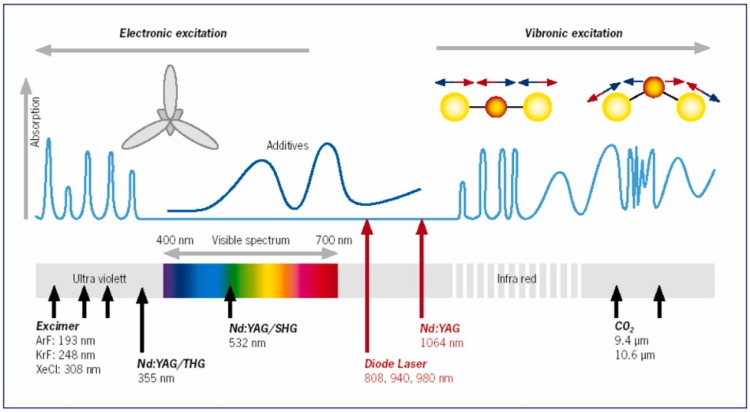
Absorption characteristics of polymer materials across different laser wavelength sources [52]. (Figure is available under Open Access).

A useful way to frame polymer design for AM is to start from the process-specific feedstock form. Vat photopolymerization uses liquid photoresins, material extrusion uses thermoplastic filaments or viscous inks, and powder bed fusion uses thermoplastic powders [38,47]. These classes are not interchangeable, because their success depends on different governing properties: resin reactivity and optical attenuation in vat systems, melt rheology and interlayer diffusion in extrusion, and powder morphology, thermal response, and coalescence in powder-bed processes. The most useful polymer designs therefore align molecular architecture, chain mobility, and phase behavior with the time scale and energy delivery mechanism of the chosen printer [45,48,49,50,51]. Interlayer bonding is the primary factor limiting the mechanical performance of fused filament fabrication (FFF) components, as insufficient polymer chain diffusion and entanglement between adjacent filaments result in weak interfacial regions. To enhance bonding through localized laser heating, efficient absorption of laser radiation by the polymer is essential [52]. Figure 2 illustrates the wavelength-dependent absorption behavior of polymers in relation to common industrial laser sources, highlighting that most unmodified thermoplastics exhibit little absorption in the visible and near-infrared regions but absorb strongly in the infrared range. This relationship is fundamental for selecting suitable laser systems and absorption-enhancing additives in laser-assisted FFF processes aimed at improving interfilament cohesion and mechanical properties.

### 2.2. Interlayer Welding and Mechanical Anisotropy

For material extrusion, including fused filament fabrication (FFF), the central design challenge is the formation of strong welds between adjacent rasters and layers. The literature consistently shows that weak interlayer bonding is the main reason for the mechanical anisotropy of FFF parts, and that strong bonds require molecular diffusion and entanglement across the interface rather than simple neck growth [52,53,54]. Because bond formation occurs only while the polymer remains hot enough for chain mobility, material selection must be co-designed with liquefier temperature, interlayer time, raster strategy, and cooling conditions. Recent studies and reviews show that lower feedstock molecular weight and higher liquefier temperature can improve bond strength, while excessive temperature can accelerate thermal degradation and weaken the final part [25,53,55,56,57].

### 2.3. Role of Crystallization in Dimensional Stability and Performance

Polymer crystallization is equally important in extrusion-based AM because it governs both dimensional stability and mechanical performance. Amorphous polymers are often preferred when dimensional accuracy is the priority, whereas semicrystalline polymers can provide higher stiffness and heat resistance but also exhibit greater shrinkage, warpage, and interlayer stress during cooling [58,59]. Reviews of polymer AM note that low crystallinity, slow crystallization kinetics, and low thermal expansion are desirable for extrusion when semicrystalline materials are used, and heated build plates or heated chambers are often needed to reduce thermal gradients. Experimental studies on PLA have further shown that porosity, crystallinity, and print orientation strongly influence tensile strength, confirming that part performance cannot be predicted from chemistry alone [60,61,62,63].

### 2.4. Rheological Requirements for Printability and Bonding

Rheology is the other key lever in extrusion processing. The printability of a filament or melt depends on whether it can be delivered through the nozzle, hold its shape after deposition, and still retain enough chain mobility for interlayer welding [64,65]. Reviews of material extrusion emphasize that polymer rheology must be understood together with print physics, because melt viscosity, shear thinning, and relaxation behavior affect flow through the nozzle, bead geometry, and bonding between deposited roads [66,67]. In this sense, polymer design for FFF is partly an exercise in balancing processability and weldability: too viscous, and extrusion becomes unstable; too fluid, and shape retention and dimensional control suffer [68,69,70,71].

### 2.5. Vat Photopolymerization: Resin Chemistry and Network Formation

Vat photopolymerization places a different set of demands on polymer chemistry. In SLA, DLP, LCD, and related systems, the resin must photopolymerize rapidly enough to build each layer, yet remain fluid enough to spread uniformly across the vat [72,73]. Studies on acrylate-based printing show that resin formulation affects every stage of the process, from exposure conditions to final mechanical properties. The working-curve framework used in vat photopolymerization links exposure energy to cure depth through intrinsic resin parameters, and thus provides a direct way to tune printability by adjusting photochemistry rather than only printer settings [74,75,76].

Recent research has shifted from merely improving printing parameters toward engineering resin chemistries that directly address the intrinsic limitations of vat photopolymerization. These material-centered strategies can be broadly classified into four complementary categories: (i) advanced photoinitiator systems, (ii) oxygen-tolerant polymerization mechanisms, (iii) shrinkage-mitigating resin formulations, and (iv) dynamic covalent network architectures.

Visible-light-responsive photoinitiators represent one of the most important developments in recent years. Compared with conventional UV-activated photoinitiators, visible-light systems offer deeper light penetration, lower phototoxicity, reduced material degradation, and improved compatibility with biological materials [77,78]. New generations of phosphine oxide derivatives, phenothiazine-based oxime esters, charge-transfer complexes, and photobase generators have enabled rapid polymerization under blue or green light while simultaneously reducing oxygen inhibition through improved radical generation efficiency. These systems are particularly attractive for high-resolution stereolithography, digital light processing, and bioprinting applications where gentle curing conditions are essential [79].

To further suppress oxygen inhibition, researchers have explored oxygen-tolerant initiation mechanisms beyond conventional free-radical polymerization. Cationic photopolymerization, thiol–ene and thiol–acrylate click reactions, and hybrid radical/cationic systems exhibit substantially lower sensitivity to atmospheric oxygen because propagation either proceeds through step-growth mechanisms or relies on photo-generated acids rather than oxygen-sensitive radicals [80,81]. These approaches shorten induction periods, increase conversion, and improve interlayer adhesion without requiring inert processing environments.

Another active area of research focuses on reducing polymerization shrinkage and residual stress. Material strategies include the incorporation of ring-opening monomers, hyperbranched oligomers, flexible urethane-acrylate segments, thiol-containing monomers, and low-shrinkage reactive diluents that reduce volumetric contraction during network formation [82,83,84]. Simultaneously, optimized oligomer-to-monomer ratios, controlled crosslink density, and tailored curing kinetics help minimize residual stresses while maintaining sufficient mechanical performance and dimensional accuracy. These developments improve both structural integrity and long-term reliability of printed components [85,86].

Perhaps the most significant recent advance is the emergence of photocurable dynamic covalent network (DCN) resins. Unlike conventional permanently crosslinked thermosets, DCNs contain reversible covalent bonds capable of bond exchange reactions under thermal, chemical, or photochemical stimulation [87]. Dynamic chemistries based on transesterification, imine exchange, disulfide exchange, boronic esters, Diels–Alder reactions, and vitrimer-type networks enable stress relaxation during curing while simultaneously providing self-healing, reprocessability, recyclability, and extended service life [88]. Consequently, DCN photopolymers are increasingly regarded as a promising route toward sustainable vat photopolymerization because they combine high printing resolution with circular-material capabilities that are difficult to achieve using conventional photocurable thermosets [89].

Collectively, these developments demonstrate that modern vat photopolymerization is increasingly driven by rational resin engineering rather than printer hardware alone. Future photocurable materials will likely integrate oxygen-tolerant initiation, visible-light activation, adaptive dynamic covalent chemistries, and multifunctional additives within single resin formulations to simultaneously improve printability, mechanical performance, durability, and sustainability [90].

### 2.6. Molecular Architecture and Resin Formulation

Within vat photopolymerization, molecular architecture is a major determinant of performance. Low-molecular-weight oligomers usually lower resin viscosity because they reduce chain entanglement, but they can also give networks with lower glass-transition temperature and poorer mechanical properties [91,92]. By contrast, higher-molecular-weight and higher-functionality oligomers can produce more highly crosslinked networks with better thermal stability, but they often become too viscous to process without reactive diluents [93,94]. This is why resin design often combines oligomers, monomers, photoinitiators, and sometimes additives or diluents: the formulation must simultaneously satisfy optical penetration, cure kinetics, and mechanical targets [79,90,95].

### 2.7. Oxygen Inhibition, Shrinkage, and Post-Curing Effects

Oxygen inhibition and shrinkage are two of the most important limitations in photopolymer design. In free-radical systems, oxygen scavenges radicals, which can slow polymerization, extend induction times, reduce conversion, and leave tacky surfaces; this effect also influences interlayer adhesion in bottom-up systems [96,97]. At the same time, excessive shrinkage can lead to distortion, delamination, and residual stress, so the formulation must balance rapid cure against volumetric stability. Post-curing is often necessary because it increases conversion, modulus, and glass-transition temperature, but it can also introduce bending or shape distortion, especially in thin parts or complex geometries [98,99,100].

### 2.8. Advances in Photoinitiators and Multi-Material Printing

Advances in photoinitiator chemistry have broadened the design space of vat photopolymerization. Reviews of advanced photoinitiating systems report that new initiators can accelerate printing, enable visible-light processing, and support new functionalities in printed parts, while still addressing the usual limitations of resolution and mechanical performance [90,101,102,103]. These studies on vat photopolymerization also show that multi-material printing is becoming more practical because material switching times have been reduced, opening the door to spatially graded properties and functional devices. These developments illustrate that resin chemistry is now used not only to make printing possible, but also to encode performance gradients and multifunctionality directly into the build.

### 2.9. Powder Properties and Processability

Powder bed fusion requires a third materials strategy centered on powder-state control. In polymer powder bed fusion, the feedstock must recoat smoothly, absorb and redistribute thermal or optical energy appropriately, and then coalesce before solidifying through cooling and crystallization [45,104]. The works in the field [49,105] emphasize that powder properties are “parent” properties for the process, because particle size, shape, flowability, packing behavior, and thermal response all govern printability and final part quality. Although early work relied heavily on empirical screening rules such as the stable sintering region and energy melt ratio, recent reviews argue that PBF materials should be developed from the standpoint of intrinsic polymer science rather than only by process trial and error [45].

### 2.10. Crystallization-Controlled Coalescence and Material Expansion

**Figure 3:**
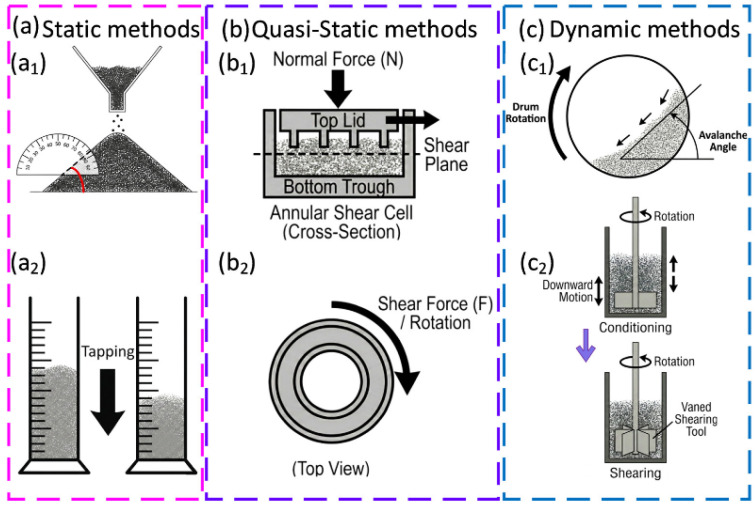
Methods commonly employed to evaluate the flow behavior of powders for PBF: (**a**) static techniques, including (**a1**) angle of repose and (**a2**) bulk and tapped density measurements; (**b**) quasi-static approaches utilizing shear testing, shown as (**b1**) cross-sectional and (**b2**) top-view configurations; and (**c**) dynamic characterization methods, including (**c1**) dynamic angle of repose and (**c2**) powder rheometry [104]. (Figure is available under Open Access).

The role of crystallization is especially pronounced in powder bed fusion. During coalescence, particles must fuse before the material crystallizes to a degree that arrests flow, and the final cooling history determines whether the part densifies without excessive distortion [106,107]. Higher crystallinity can improve stiffness and thermal resistance, but it also increases shrinkage, which raises the risk of warpage and residual stress. For that reason, polymer PBF has historically favored semicrystalline materials such as polyamides, while newer studies aim to expand the material portfolio to engineering polymers such as PEEK, PEK, PPS, PBT, polypropylene, and polyethylene through careful control of powder morphology and thermal behavior [104,108,109]. Since uniform powder spreading directly affects layer quality, density, and defect formation, reliable assessment of powder flow behavior is essential before processing. Various characterization methods have therefore been developed to evaluate powder performance under different stress and motion conditions, ranging from simple static measurements to advanced dynamic rheological analyses [104]. Figure 3 summarizes the principal techniques used to characterize the flow behavior of polymer powders for PBF, highlighting the complementary information provided by static, quasi-static, and dynamic testing approaches.

### 2.11. Unified Perspective: Toward Process-Matched Polymer Design

Taken together, the fundamentals of polymer design for AM can be summarized as a process-matched control of molecular weight, architecture, functionality, crystallization, rheology, and phase behavior [38,48]. In extrusion, the priority is chain mobility and weld formation; in vat photopolymerization, it is resin reactivity, cure depth, and network formation; and in powder bed fusion, it is powder flow, heat transfer, and crystallization-controlled coalescence [25,110]. The field is moving away from generic “printable polymer” screening toward a structure–process–property approach in which the polymer is intentionally designed for the thermal, optical, and mechanical time scales of the printer. That shift is what will ultimately turn polymer AM from a prototyping tool into a robust manufacturing platform [13,38,111].

## 3. Classes of Functional Polymers for Additive Manufacturing

### 3.1. Conductive and Electrically Active Polymers

Conductive and electrically active polymers (CPs) represent one of the most dynamically evolving material classes in additive manufacturing (AM), combining the processing versatility of polymers with the electronic functionality of metals or semiconductors [112]. A growing variety of AM technologies are now being applied to CPs, including vat photopolymerization, material extrusion, powder bed fusion, and material jetting, each offering distinct trade-offs between resolution, throughput, and material compatibility [113].

Among the most widely studied intrinsically conductive polymers for AM is poly(3,4-ethylenedioxythiophene):poly(styrenesulfonate) (PEDOT:PSS), which has attracted exceptional interest due to its tissue-like mechanical properties, tunable conductivity, and biocompatibility [114]. Advances in ink formulation have allowed PEDOT:PSS to be processed via direct ink writing (DIW), with ionic liquid additives enabling simultaneous on-demand biocompatibility, structural integrity, and high conductivity—overcoming longstanding barriers associated with post-treatment steps and aspect-ratio printing limitations [115]. In a complementary approach, conformal wearable bioelectronics have been fabricated by depositing PEDOT:PSS/PVA composite bio-inks directly onto the complex surface of human skin using DIW, demonstrating the feasibility of in situ printed sensors for physiological motion monitoring [116].

Beyond intrinsically conductive systems, CP-filled polymer composites constitute a second major family. Conductive fillers—including carbon black, carbon nanotubes, graphene, and metallic nanoparticles—are dispersed within thermoplastic matrices to create percolating networks that enable continuous electron transport while preserving printability [31]. The introduction of dual-nozzle and multi-material fused deposition modeling (FDM) printers has further expanded possibilities, allowing the simultaneous deposition of conductive and insulating polymers to fabricate integrated electronic circuits in a single build step without post-assembly [117]. The resulting materials are finding use in wearable health-monitoring devices, electromagnetic interference shielding, energy storage components, and implantable bioelectronic interfaces, illustrating the breadth of impact achievable through conductive polymer AM [118].

Light-based multi-material 3D printing has also been applied to PEDOT:PSS, enabling the fabrication of micro-structured, bio-shaped electrodes with dry adhesive properties for bioelectronic applications using digital light processing (DLP)—a route that affords sub-millimetre resolution and seamless integration with biological surfaces [119].

### 3.2. Stimuli-Responsive Polymers

**Figure 4:**
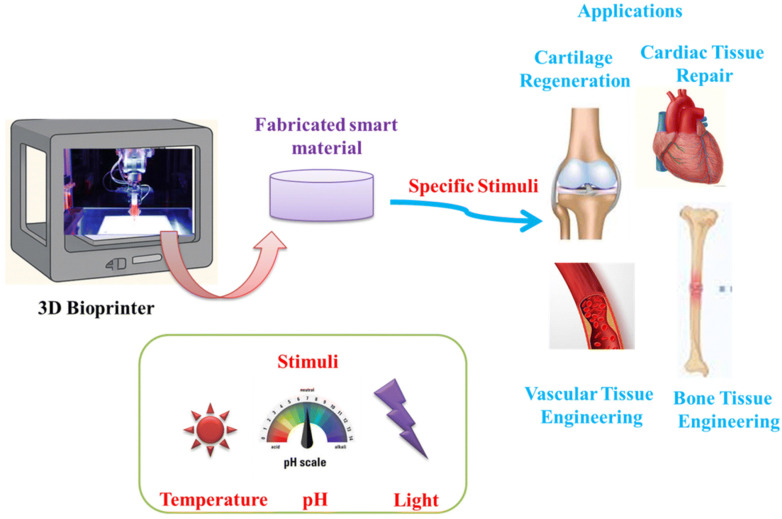
4D printing uses intelligent biomaterials that can alter their shape and functionality in response to external stimuli, offering significant potential for applications in regenerative medicine [121]. (This figure is available under Open Access).

Stimuli-responsive polymers—materials capable of undergoing programmable, reversible changes in shape, stiffness, or function in response to external triggers such as heat, light, pH, moisture, or magnetic fields—have emerged as the foundational class for four-dimensional (4D) printing [120]. By incorporating the dimension of time into the AM workflow, these materials enable the fabrication of dynamic structures that evolve after printing, unlocking applications in soft robotics, deployable structures, biomedical implants, and drug delivery [121]. Figure 4 illustrates the fundamental concept of 4D bioprinting, where smart, stimuli-responsive biomaterials enable printed constructs to undergo programmed, time-dependent changes in shape and/or function. Unlike conventional 3D-printed structures, these materials respond to external cues such as temperature, pH, light, or humidity, allowing dynamic adaptation after fabrication. This stimulus-driven transformation underpins the core advantage of 4D printing in regenerative medicine, enabling the development of biomimetic, adaptable systems that better replicate the behavior of native tissues and support advanced applications in tissue engineering and therapeutic delivery.

Shape-memory polymers (SMPs) are among the most studied stimuli-responsive materials for AM. They are programmed to hold a temporary shape and recover their permanent geometry upon application of a stimulus, most commonly heat [122]. Smart implants that adapt to changing physiological conditions over time represent a compelling application area; 4D-printed shape-memory thermomorphs are being investigated for spinal cages, cardiovascular stents, and other minimally invasive devices that self-deploy after introduction into the body [123]. Hydrogels based on poly(acrylic acid) have been 4D-printed to concurrently exhibit shape-memory and self-healing properties, with their reversible strong-to-weak gel transition occurring near human body temperature (37 °C), suggesting utility in personalised biomedical devices [124].

Liquid crystal elastomers (LCEs) constitute a particularly powerful sub-class of stimuli-responsive polymers for AM, as the alignment of mesogenic units along the print path—achieved through nozzle shearing and tensile action during DIW extrusion—can be directly programmed to produce anisotropic actuation upon heating [125]. Recent work has demonstrated 4D-printed LCE lattices with thermally programmable deformation sequences achievable through multi-material printing strategies, enabling spatially graded actuation responses [126]. Photoresponsive LCE composites incorporating light-driven molecular motors have further expanded the stimulus palette, permitting untethered, wireless actuation by visible or UV light [127].

Chemical stimuli-responsive hydrogels represent another important category, with pH- and reactive-oxygen-species-sensitive formulations compatible with DLP 4D printing demonstrating promise for targeted drug delivery and tissue engineering [128]. Trigger-free shape-memory hydrogel substrates fabricated by digital 4D printing—where spatiotemporal control of light exposure programs pixelated polymer network gradients—enable customised shape-shifting with predefined onset periods, relevant for minimally invasive conformable bioelectronic implants [129].

### 3.3. Elastomers and Soft Materials

Elastomers and soft materials for AM such as silicone elastomers and thermoplastic polyurethanes (TPUs) are characterised by low elastic moduli (typically below 1 MPa for silicones), large reversible deformations, and mechanical properties resembling biological tissues—attributes that render them indispensable for soft robotics, wearable electronics, prosthetics, and medical devices [130].

Silicone elastomers have been processed primarily via DIW and stereolithography. Their inherently low viscosity has historically posed processability challenges; however, the addition of nano-silica as a rheology modifier has been shown to improve printability without compromising the outstanding stretchability of the base material [131]. Beyond structural applications, DIW of silicone elastomers has been applied to the fabrication of microfluidic devices and soft robotic components, establishing a new standard in the field and demonstrating the versatility of the approach across length scales [132]. Comprehensive reviews of additive-manufactured soft robotics have catalogued a diverse range of silicone-based actuator architectures—from sensorised compliant fish-tails to pneumatic humanoid hands—fabricated using multi-material embedded 3D printing strategies [130].

TPU, a block copolymer with alternating hard (isocyanate) and soft (polyol) segments, occupies a central position among printable elastomers by bridging the gap between rigid plastics and silicone rubbers [133]. Shore hardness values spanning from 60A to 80D can be achieved by adjusting the hard-to-soft segment ratio, and both FDM and selective laser sintering (SLS) have been applied to process TPU into functional components [134]. In soft robotics, TPU pneumatic networks (pneu-nets) and monolithic soft grippers have been printed using dual-extruder printers that simultaneously deposit TPU grades of different hardness (e.g., 95A for rigid regions and 80A for extensible regions) to achieve spatially differentiated stiffness and airtight embedded connectors [130]. TPU-based 3D-printed pressure sensors, prosthetic fingers, and exosuit components have been reported for wearable human–robot interaction, highlighting the material’s suitability for conformal devices that must endure repeated mechanical loading [135].

### 3.4. High-Performance Polymers

High-performance polymers (HPPs) and ultra-performance polymers (UPPs)—including polyetheretherketone (PEEK), polyetherketoneketone (PEKK), and polyimide (PI)—are being increasingly integrated into AM workflows to address applications where standard thermoplastics cannot meet demands for thermal stability, chemical resistance, or mechanical performance [136]. Material extrusion-based AM (MEXAM) has emerged as the primary platform for processing these materials, capitalising on their high strength-to-weight ratios, performance retention under harsh conditions, and compatibility with industrial FDM hardware equipped with nozzle temperatures exceeding 400 °C [137].

PEEK has stood out as the leading HPP for AM, particularly in biomedical and aerospace applications. Its exceptional biocompatibility, bone-analogous elastic modulus (~4 GPa), and radiolucency have made it the preferred alternative to metallic implants for orthopaedic, spinal, and craniofacial reconstruction [138]. A revolution in implant manufacturing has been enabled by patient-specific 3D-printed PEEK components, with mechanical performance depending critically on FFF parametrisation—layer height, print speed, nozzle temperature, and build orientation—as well as post-processing thermal annealing treatments that enhance crystallinity and interlayer adhesion [139,140]. Additive-manufactured PEEK has also been evaluated in low-pressure (space-representative) environments, demonstrating that vacuum conditions can improve interlayer bonding in PEEK and ULTEM 9085 specimens, opening prospects for in-space manufacturing of structural components [141].

PEKK, a PAEK-family polymer with a slower crystallisation rate than PEEK, offers greater processing flexibility, reduced warping, and improved z-strength in FDM, making it particularly attractive for complex aerospace and defence parts requiring precise thermal performance [142,143]. Polyetherimide (PEI, commercially available as ULTEM 9085 and ULTEM 1010) combines high-temperature resistance, excellent flame retardancy (UL94 V-0), and dimensional stability, and holds FAA NCAMP certification datasets enabling flight-qualified part production for commercial aircraft programmes [144]. The global polymer AM equipment market—driven by growing adoption of HPPs—was valued at approximately USD 320 million in 2024 and is projected to reach USD 1.55 billion by 2032, underscoring the commercial momentum behind these materials [145].

Beyond material selection, post-processing and consolidation technologies have become equally important for enabling high-performance polymers in demanding aerospace and space applications [146,147]. Additively manufactured PEEK, PEKK, PEI, and related polyaryletherketone (PAEK) components typically contain residual stresses, incomplete interlayer diffusion, and localized porosity that can compromise long-term structural reliability under cyclic thermal and mechanical loading. Consequently, modern processing workflows increasingly integrate controlled post-treatment operations to optimize the final microstructure [58,148].

Vacuum thermal annealing has emerged as one of the most effective approaches for high-performance thermoplastics. Carefully controlled annealing above the glass transition temperature but below the melting point promotes secondary crystallization, increases crystalline perfection, relieves residual stresses, and enhances interlayer molecular diffusion without inducing significant geometric distortion [149,150]. Performing this treatment under vacuum further minimizes thermo-oxidative degradation and moisture uptake while improving dimensional stability, making it particularly attractive for aerospace structures and in-space manufacturing where components experience large thermal fluctuations and reduced atmospheric pressure [151,152]. Experimental studies have demonstrated measurable improvements in tensile strength, fracture toughness, creep resistance, and thermal stability following optimized vacuum annealing of FFF-printed PEEK and PEKK components [153,154].

Continuous thermo-mechanical consolidation has also become an important strategy for improving structural integrity during or immediately after deposition. Infrared-assisted heating combined with continuous hot-roller compaction maintains the deposited filament above its crystallization temperature for longer periods while simultaneously applying compressive pressure that reduces void formation and promotes intimate fiber–matrix contact [155,156,157]. For continuous carbon-fiber-reinforced PEEK composites, this approach substantially improves interlaminar shear strength, decreases porosity, and produces more homogeneous crystallinity throughout the laminate. Similar consolidation strategies are increasingly incorporated into automated additive manufacturing systems for large aerospace structures, where maintaining consistent interlayer quality is essential for fatigue resistance and long-term durability [158,159].

For future extraterrestrial manufacturing, these post-processing technologies are expected to become even more critical. Components fabricated under reduced-gravity or vacuum environments experience different heat-transfer conditions than terrestrial printing, making integrated thermal management and consolidation essential for achieving predictable crystallization behavior, minimizing residual stresses, and ensuring reliable mechanical performance [160,161]. Consequently, next-generation processing workflows increasingly combine optimized printing parameters with in situ thermal monitoring, infrared consolidation, hot-roller compaction, and controlled post-print annealing to produce flight-qualified polymer structures suitable for aerospace, lunar, and deep-space missions [162,163,164].

### 3.5. Bio-Based and Biodegradable Polymers

Bio-based and biodegradable polymers are gaining prominence in AM as the field responds to growing imperatives for environmental sustainability and circular economy alignment. Derived from renewable biomass sources—including corn starch, sugarcane, and microbial fermentation—these materials offer degradation pathways into non-toxic byproducts, reducing end-of-life environmental burden relative to petroleum-based counterparts [165].

Poly(lactic acid) (PLA) is the most widely deployed bio-based polymer in FDM due to its renewability, processability, and biodegradability. It degrades in vivo into non-toxic byproducts over periods ranging from 6 months to several years, and its high stiffness makes it useful for fixation devices and tissue engineering scaffolds [166]. Compositing PLA with calcium phosphate ceramics—such as β-tricalcium phosphate (β-TCP) or bioglass—improves its osteoinductive potential and mechanical strength, with PLA-bioglass composites demonstrating up to 80% improvement in mechanical strength alongside confirmed in vitro and in vivo biocompatibility [167]. Despite these advances, PLA’s brittleness and limited thermal stability remain constraints that motivate ongoing copolymerisation and blending strategies to balance stiffness, toughness, and osteogenic performance [168].

**Figure 5:**
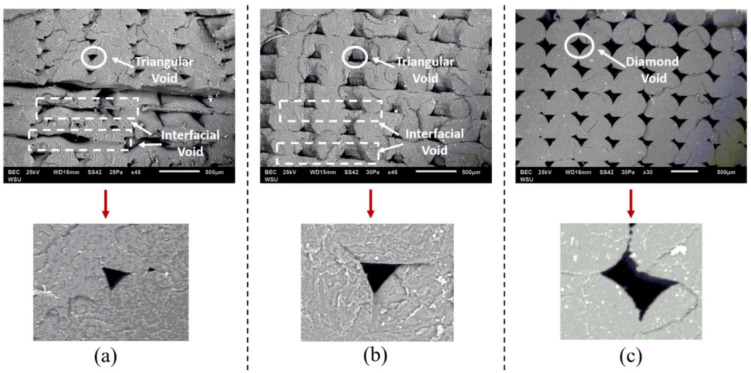
SEM characterization of void development in FFF-fabricated PLA samples at a raster angle of 0°, nozzle temperature of 210 °C, and feed rate of 50 mm/s, with layer thicknesses of (**a**) 0.2 mm, (**b**) 0.3 mm, and (**c**) 0.4 mm [169]. (Figure is available under Open Access).

In [169], the authors experimentally investigated the influence of key fused filament fabrication (FFF) process parameters on the mechanical performance and microstructural characteristics of polylactic acid (PLA) components. Figure 5 presents scanning electron microscopy (SEM) images illustrating void formation in fused filament fabrication (FFF)–printed PLA specimens under controlled processing conditions (raster angle 0°, nozzle temperature 210 °C, and feed rate 50 mm/s), with varying layer thicknesses of 0.2, 0.3, and 0.4 mm. These micrographs provide detailed insight into the internal defect morphology, highlighting how increasing layer thickness influences the size, distribution, and geometry of voids within the printed structure.

Polyhydroxyalkanoates (PHAs), including polyhydroxybutyrate (PHB) and its copolymers, represent a fully bio-derived, home-compostable family of polyesters produced by microbial fermentation [170]. PHAs are increasingly adopted in AM through FDM and related extrusion processes; PHA blends optimised via a Design of Experiments approach have demonstrated elongation at break exceeding 2000%—far surpassing PLA at 29%—and minimal warping when crystallinity is maintained below 18%, underscoring their potential for flexible, biodegradable functional parts [171]. In the biomedical domain, PHAs are being evaluated for drug delivery systems, resorbable sutures, and tissue engineering scaffolds, with their favourable degradation kinetics and non-toxic breakdown products distinguishing them from synthetic biodegradable competitors [172].

Despite their promise, the market share of bio-based and biodegradable polymers (excluding polysaccharides) remained at only approximately 0.2% of the global plastics production capacity in 2023, reflecting persistent cost and performance challenges [173]. Bridging these gaps through metabolic engineering of microbial production strains, reactive extrusion blending with natural fillers, and further AM process optimisation will be critical to enabling widespread industrial adoption of these environmentally responsible material classes [174].

## 4. Polymer Nanocomposites in Additive Manufacturing

### 4.1. Reinforcement with Nanoparticles and Fibers

The integration of nanoscale and microscale reinforcements into polymer matrices has become one of the most fertile research directions in additive manufacturing (AM), enabling simultaneous improvements in mechanical, thermal, and functional performance. Incorporating nanoparticles, carbon fibers, and natural fibers into polymer matrices enhances mechanical, thermal, electrical, and multifunctional properties, offering the potential to fabricate lightweight, high-strength, and geometrically complex components with tailored functionalities for aerospace, automotive, biomedical, and electronics applications [175]. The global market for polymer nanocomposites underscores this momentum: valued at approximately USD 5.5 billion in 2023, it is projected to reach USD 21.8 billion by 2030 at a compound annual growth rate of 21.6%, with nanoclay and carbon nanotube composite segments showing especially steep growth [28].

Ceramic nanoparticles are among the most extensively studied fillers for material-extrusion printing. Silicon nitride (Si_3_N_4_) nanoparticles dispersed in ABS at the optimum 4 wt.% loading delivered a 30.3% increase in flexural strength and a 47.2% increase in flexural toughness compared with neat ABS, with rheological and processability assessments confirming that the melt-viscosity increase remained within acceptable bounds for fused filament fabrication [176]. Similarly, titanium nitride (TiN) nanoparticles in ABS reached their optimum at 6 wt.%, yielding a 42.3% gain in flexural modulus of elasticity and a 54.0% gain in toughness; atomic force microscopy and scanning electron microscopy revealed a direct correlation between TiN surface concentration and mechanical response [177]. Polycarbonate reinforced with aluminium nitride (AlN) at 2 wt.% achieved 32.8% higher tensile strength and 51.6% higher toughness compared with the unfilled matrix, establishing ceramic nitride nanoparticles as a compelling class of fillers for thermoplastic extrusion AM [178].

**Figure 6:**
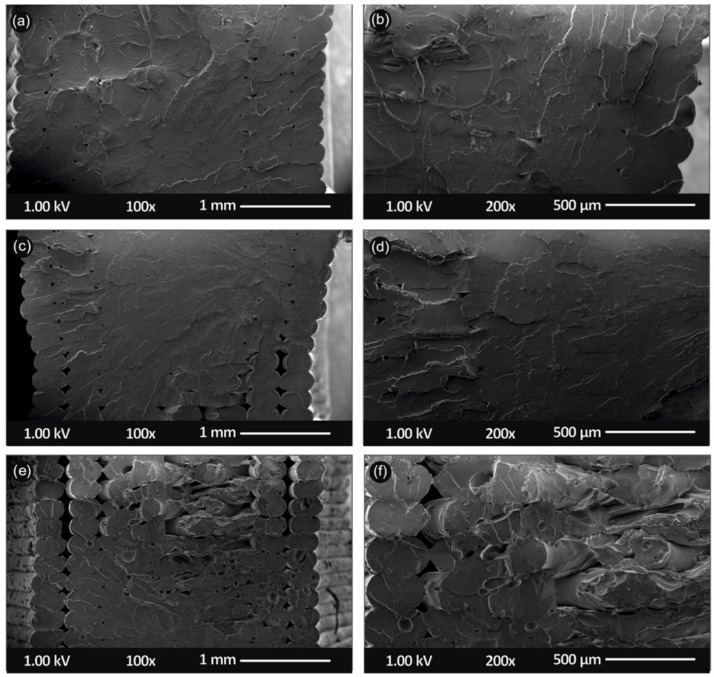
SEM micrographs depicting the fracture surface morphology and microstructural features of the tensile-tested 3D-printed specimens at two magnification levels are shown. All samples were fabricated with a layer thickness of 300 µm. Images at 100× and 200× magnifications correspond to (**a**,**b**) neat PLA, (**c**,**d**) PLA/SiO_2_ with 1.0 wt.% filler, and (**e**,**f**) PLA/SiO_2_ with 4.0 wt.% filler, respectively [179]. (Figure is available under Open Access).

Oxide nanoparticles have been equally productive. SiO_2_ nanoparticles in PLA at loadings up to 1 wt.% improved overall mechanical strength and imparted antimicrobial activity against Escherichia coli and Staphylococcus aureus, while thermogravimetric analysis confirmed enhanced thermal stability [179]. Hollow SiO_2_ nanoparticles incorporated into a Bis-GMA/TEGDMA photopolymer resin via DLP printing promoted phonon scattering that markedly reduced thermal conductivity and diffusivity, demonstrating that nanoparticle architecture—not merely chemistry—determines thermal outcomes; above 5 wt.%, however, agglomeration caused a non-linear decrease in mechanical performance, foreshadowing the loading-ceiling problem common to all nanoparticle systems [180]. In material-extrusion AM, copper nanoparticles (Cu-NPs) introduced into PLA and TPU filaments provided antibacterial function through surface-localised Cu^0^, Cu^+^, and Cu^2+^ species, although X-ray photoelectron spectroscopy revealed that FFF processing substantially depleted these surface species, highlighting how extrusion thermal cycles can modify nanoparticle chemistry and reduce anticipated functionality [181]. The study [179] reports an experimental investigation on FFF 3D-printed PLA/SiO_2_ nanocomposites, examining how silica nanoparticle loading influences mechanical behavior and microstructural characteristics through systematic fabrication and characterization. Figure 6 presents scanning electron microscopy (SEM) images of the fracture surfaces after tensile testing for pure PLA and PLA/SiO_2_ nanocomposites (1.0 wt.% and 4.0 wt.%), captured at 100× and 200× magnifications. The figure highlights differences in fracture morphology, revealing a more brittle fracture behavior in neat PLA, while the nanocomposites exhibit comparatively more ductile features. Additionally, the micrographs confirm a homogeneous internal structure without significant defects across all samples, indicating good filament quality and effective dispersion of SiO_2_ nanoparticles within the PLA matrix.

Naturally derived nanofillers offer bio-compatibility alongside reinforcement. Halloysite nanotubes (HNTs) functionalised with pimelic acid and compounded into polypropylene at 0.5–1.5 wt.% produced filaments with a tunable stiffness–impact balance; infrared spectroscopy confirmed interfacial bonding between the acid-modified halloysite surface and the PP matrix, while the 1.0 wt.% formulation represented the optimum trade-off between these competing mechanical properties [182]. Boron nitride (BN)-filled polymer composites at 20 wt.% printed by vat photopolymerization achieved a 215% increase in tensile strength while retaining flexibility, with feature resolutions of 125 µm and infrared thermography confirming enhanced heat transfer under simulated operating conditions—a combination that makes BN a compelling thermally conductive, electrically insulating filler for printed electronics housings [183].

Fiber reinforcement, whether short or continuous, introduces mechanically anisotropic performance that nanoparticles alone cannot match. In short carbon fiber (SCF)/polypropylene composites fabricated by FDM, tensile strength increased by up to 35% and bending strength by approximately 40% compared with pure PP; the SCF network simultaneously mitigated the warping typical of PP cooling and established electrical conductivity that scaled linearly with fiber content up to 70 °C [184]. The principal microstructural challenge in short-fiber FDM is that molten-matrix flow aligns fibers predominantly along the printing path, generating pronounced stiffness and strength anisotropy: properties in the longitudinal build direction substantially exceed those in the transverse direction, a discrepancy that must be addressed in both simulation and structural design [185].

Continuous fiber-reinforced thermoplastic (CFRTP) printing elevates mechanical performance further but introduces new process complexities. A review of continuous carbon fiber/thermoplastic AM identifies robust fiber–matrix adhesion as the primary challenge: inadequate interfacial bonding induces delamination and progressive performance degradation, and surface pre-treatments such as dopamine coating combined with post-processing compression have been shown to raise flexural strength by 27% and interlaminar shear strength (ILSS) by 172% relative to untreated specimens [186]. For continuous carbon fiber/PEEK systems, achieving adequate interlayer adhesion requires a mold-conformal infrared heating module with dual hot rollers to provide continuous thermo-mechanical consolidation during deposition—an approach that yields high-quality structures with rotational geometries but demands specialised hardware beyond standard desktop FDM platforms [187].

### 4.2. Graphene, Carbon Nanotubes, and MXenes

Among all nanofillers explored for polymer AM, two-dimensional (2D) carbons—graphene and its derivatives—and the emerging family of 2D transition-metal carbides and nitrides known as MXenes, together with quasi-1D multi-walled carbon nanotubes (MWCNTs), have attracted the greatest research effort. These materials combine exceptional intrinsic electrical conductivity, nanoscale stiffness, and large specific surface area with a potential for multifunctional performance that simpler inorganic fillers cannot replicate.

Graphene nanoplatelets (GNPs) reinforcing PLA via FDM at just 0.5 wt.% produced a 22.7% improvement in tensile strength, a 32.3% increase in flexural strength, and raised the thermal decomposition onset to 320 °C, while TEM confirmed uniform nanosheet dispersion and adequate interfacial bonding within the matrix [188]. When GNP content was raised to 5 wt.%, the benefits scaled substantially: tensile strength improved by 67%, Young’s modulus increased by 205%, and hardness by 44% relative to neat PLA; machine-learning models (XGBoost, Gaussian Process Regression) accurately predicted these outcomes as a function of nozzle temperature, print speed, layer thickness, and build orientation, pointing the way toward data-driven formulation optimisation [189]. Hybrid bi-filler PLA composites combining GNPs and MWCNTs at a 3%/3% ratio exhibited a synergistic conductivity enhancement beyond what either mono-filler system could achieve at equivalent total loading, because the complementary geometries—2D platelets and 1D tubes—build interpenetrating conductive networks with greater percolation efficiency [190].

In FDM of ABS/MWCNT composites, selective alignment of nanotubes along the printing direction via flow-induced orientation raised electrical conductivity from 6.88 × 10^−2^ S/m in the raw compound to 1.19 × 10^1^ S/m in a single printed filament, and caused a directional anisotropy in printed samples: conductivity parallel to the print direction (1.22 S/m) was more than 16 times higher than the transverse value (7.34 × 10^−2^ S/m) [191]. PLA-MWCNT nanocomposite filaments produced by an eco-friendly hybrid processing method showed increased mechanical strength, thermal stability, and structural integrity over neat PLA, with ANSYS finite-element simulation deviating from experimental tensile, compressive, and flexural values by only 4.9–6.7%, demonstrating the tractability of simulation-guided design for these systems [192].

For vat photopolymerization systems, UV-absorbing carbonaceous fillers constrain loading severely. In SLA printing of photopolymer nanocomposites, MWCNTs limited printability to just 0.05 wt.% due to a 90% reduction in UV penetration depth at 0.25 wt.%, while boron nitride nanoparticles—being UV-transparent—remained printable at up to 1.5 wt.% and outperformed MWCNTs in hardness and elastic modulus improvements at equivalent nanofiller concentration [193]. Reduced graphene oxide (rGO), whose minimal UV absorption preserves cure depth and optical clarity, can be incorporated in DLP systems at weight fractions up to ~0.35 vol% without degrading printability, making it the most photopolymerization-compatible of the common carbon 2D fillers [194].

MXenes—a growing family of 2D materials with the formula Mn + 1XnTx (M = early transition metal, X = C or N, Tx = surface terminations)—combine metallic electrical conductivity with hydrophilic, chemically reactive surfaces that facilitate aqueous colloidal processing without flammable solvents. Ti_3_C_2_T_x_ MXene incorporated into photosensitive resins for LCD-based 3D printing at 0.5 wt.% enhanced ultimate tensile and flexural strengths by 32.1% and 42.7%, respectively, and raised the glass transition and 5–weight-loss thermal degradation temperatures by 7.4 °C and 10.6 °C, attributed to hydrogen bonding between MXene surface –OH/=O terminations and the resin network [195].

Direct ink writing (DIW) of Ti_3_C_2_T_x_ MXene/waterborne polyurethane inks onto woven textiles demonstrated the ideal shear-thinning rheological behaviour required for DIW: viscosity decreasing steeply with shear rate, solid-like behaviour (G′ > G″) at rest transitioning to liquid-like flow under printing stress, enabling extrusion through narrow nozzles while maintaining post-deposition shape fidelity [196]. High-performance piezoresistive pressure sensors have been fabricated by DLP-printing of geometrically optimised polyurethane (PU) sponge architectures subsequently coated with Ti_3_C_2_T_x_ MXene; the 500 µm × 300 µm configuration achieved resistance change rates from −11.02% at 25 Pa to −163.78% at 2.25 kPa with less than 1.5% variation over 1000 measurement cycles, validating the marriage of DLP geometric precision with MXene’s electromechanical sensitivity [197].

The EMI shielding potential of MXene composites fabricated by AM is equally compelling. A review of 2D nanomaterial–polymer composites for EMI shielding concludes that MXene’s unique hydrophilicity and surface chemistry (–OH, =O, –F terminations) allow colloidal processing that preserves electrical performance, and that 3D printing enables structured architectures—layered, segregated, porous—that outperform homogeneous films of the same composition [198]. Three-dimensionally printed MXene/CNT/polyimide aerogel frames with a gradient-conductivity architecture achieved absorption-dominated EMI shielding with ultra-low reflection, directly addressing the secondary electromagnetic-radiation contamination problem inherent in highly reflective homogeneous conductors [199].

### 4.3. Property Enhancement Versus Printability Trade-Offs

The central paradox of nanocomposite AM is that the filler attributes most responsible for property gains—high aspect ratio, large specific surface area, and strong inter-particle interactions—simultaneously undermine processability. Managing this tension is not a secondary concern but a prerequisite for translating laboratory performance data into reliably printed parts.

In extrusion-based systems, nanoparticle loading raises melt viscosity; when viscosity exceeds the acceptable processing range, nozzle clogging terminates printing before the intended geometry is completed. Machine-learning analysis of extrusion AM of PLA-based nanocomposites identified complex viscosity, flow consistency index, and thermal stability as the most influential predictors of printability, while crystallisation enthalpy emerged as the dominant driver of surface roughness—a result that underscores the multivariable, coupled nature of the process [200]. A comprehensive review on nanocomposite AM notes the additional risk of nozzle wear: hard ceramic and carbon nanofillers abrade nozzle bores over repeated printing cycles, gradually degrading both dimensional accuracy and surface finish [201].

In vat photopolymerization, acceptable resin viscosity is capped at approximately 0.1–10 Pa·s; exceeding this range prevents layer recoating and introduces inter-layer defects that compromise part integrity [95]. Opaque or UV-absorbing fillers further restrict printability by attenuating cure depth: at just 0.25 wt.%, MWCNTs reduced UV penetration depth by 90%, limiting usable loading to ~0.05 wt.%—far below concentrations that would maximise electrical performance [193]. The percolation threshold concept becomes central here: carbonaceous fillers in photopolymer resins reach electrical percolation at lower concentrations than rheological percolation, creating a narrow but exploitable window in which conductivity is substantially enhanced while viscosity remains only moderately elevated and UV cure depth is still adequate [202].

Optimal filler loading follows a non-monotonic pattern across virtually all polymer/nanoparticle systems: properties improve with increasing reinforcement up to a threshold concentration beyond which agglomeration and chain-mobility disruption cause net deterioration. For SiO_2_/PLA, the threshold lay at 1 wt.%; for TiN/ABS at 6 wt.%; for hollow SiO_2_/photopolymer above 3 wt.%; and for Si_3_N_4_/ABS at 4 wt.%. Exceeding these thresholds not only reduces mechanical performance but also worsens printability through elevated viscosity and nozzle blockage, creating a doubly adverse outcome [176]. Nanographite addition to PLA filaments illustrates the microstructural origin of this ceiling: SEM of filament cross-sections showed that agglomerated nanographite particles produced irregular surface morphology and reduced filament roundness, with the 0.5 wt.% sample achieving the best roundness value of 99.02% [203].

Surface functionalisation is the most broadly applied strategy to depress the agglomeration threshold and widen the processable loading range. Advanced techniques including sonication, surface functionalisation, and compatibiliser addition enhance dispersion and boost mechanical strength and conductivity, while high-shear mixing and in situ polymerisation ensure structural integrity; however, excessive chemical treatment can itself compromise the nanoparticle’s intrinsic electronic or mechanical properties, creating a stability–performance trade-off that requires case-by-case optimisation [204].

**Figure 7:**
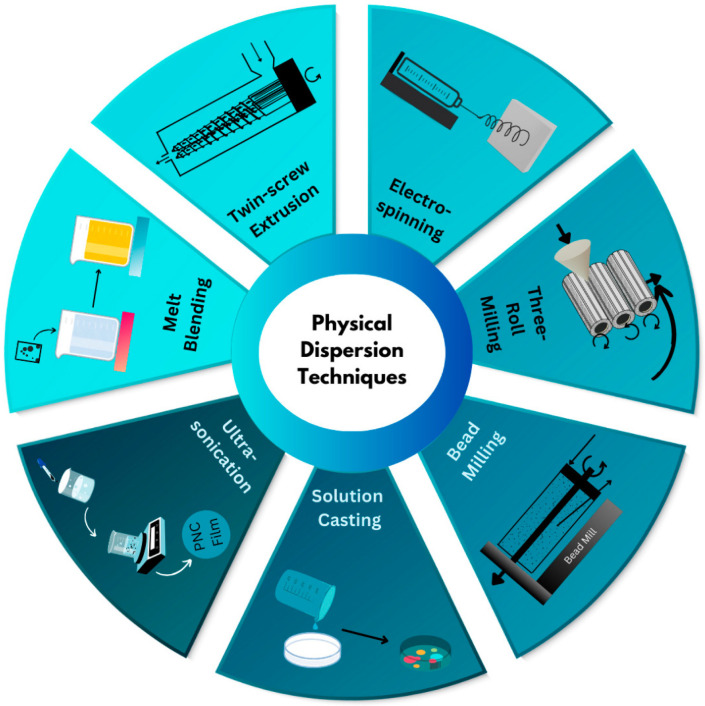
Physical Methods for Nanoparticle Dispersion in Polymer Nanocomposites [204]. (Figure is available under Open Access).

For fibrous reinforcements, printability is further complicated by fibre-induced anisotropy and by the difficulty of maintaining interlayer cohesion. In continuous-fibre AM, inadequate fibre–matrix wetting produces interlayer porosity and delamination that critically degrade out-of-plane mechanical performance; post-consolidation compression reduces void content but requires secondary process steps that increase cost and cycle time [186]. For DIW of functional nanocomposite inks, quantitative printability metrics—yield stress, storage modulus G′, shear-thinning index—must be simultaneously satisfied for extrusion to proceed through narrow nozzles (100–800 µm) while ensuring post-deposition shape retention; finite-element simulations now offer a route to predict printability windows before physical ink preparation, reducing experimental trial-and-error [205]. Figure 7 presents the major physical dispersion techniques employed in polymer nanocomposites (PNCs), including ultrasonication, bead milling, three-roll milling, twin-screw extrusion, solution casting, melt blending, and electrospinning. These methods use mechanical forces to disperse nanoparticles uniformly within polymer matrices, thereby improving nanoparticle distribution and enhancing composite performance. Uniform dispersion is essential for achieving the superior mechanical, thermal, electrical, and barrier properties that characterize advanced PNCs.

Collectively, these findings indicate that advances in nanocomposite AM require a co-optimisation philosophy in which material formulation, reinforcement surface chemistry, dispersion method, and process parameters are tuned together rather than sequentially. Emerging strategies—machine-learning-guided ink formulation, ultrasonic vibration-assisted extrusion, AI-driven real-time process monitoring—are beginning to automate this multi-variable optimisation and promise to close the gap between the exceptional properties measurable in well-dispersed laboratory specimens and the consistent performance achievable in production-scale printing [28].

## 5. Processing–Structure–Property Relationships

### 5.1. Anisotropy in Printed Parts

Additive manufacturing (AM) builds components in a layer-by-layer fashion, and this fundamental process characteristic is the primary origin of mechanical anisotropy—one of the most persistent challenges limiting the wider industrial adoption of printed parts [206]. The anisotropic response manifests differently depending on the AM technology and material system, but in every case it can be traced to two interrelated sources: the directional microstructure that develops during solidification and the geometric discontinuities introduced at layer interfaces.

In metal powder bed fusion (PBF) processes such as selective laser melting (SLM) and laser powder bed fusion (LPBF), the steep thermal gradients imposed by a rapidly traversing laser promote preferential grain growth along the build direction [207]. Grains nucleate epitaxially on previously solidified layers and elongate into columnar structures oriented parallel to the maximum heat-flow vector, which coincides with the build direction. This columnar texture carries a pronounced crystallographic signature: in austenitic alloys such as Inconel 718 and stainless steel 316L, a strong ⟨001⟩ fiber texture parallel to the build axis is routinely observed by electron backscatter diffraction (EBSD) [208]. The consequence for mechanical performance is substantial. In LPBF-processed SS316L, horizontally oriented specimens consistently show higher tensile strength than their vertically oriented counterparts for a given laser power, a difference attributable to the change in the operative grain boundary character with respect to the loading axis [209]. In a laser powder bed fusion high-entropy alloy study, horizontal specimens exhibited yield and ultimate tensile strengths approximately 15% and 21% higher, respectively, than vertical specimens, while the vertical orientation provided superior ductility. Quantitative analysis attributed the strength differential primarily to differences in dislocation density rather than texture alone [210].

For polymer-based extrusion processes such as fused deposition modelling (FDM/FFF), the origin of anisotropy shifts toward the interlayer bond. Because molten filament deposits cool rapidly, the reptation-based chain interdiffusion that creates true molecular-level bonding across adjacent beads is kinetically limited, leaving interlayer interfaces as planes of weakness [26]. Studies on FDM-printed poly(ether ether ketone) (PEEK) demonstrate that the printing path exerts a dominant influence on anisotropic behaviour: under identical build orientations, samples printed with transverse or through-thickness paths exhibited elongations at break below 5%, while other path configurations reached nearly 96% elongation, underscoring that raster angle selection is as critical as build orientation itself [211]. In a complementary investigation of FDM-printed PLA, aligning the principal axis perpendicular to the build platform yielded significantly better tensile strength and degree of conversion compared with parallel configurations [212].

Photopolymer-based processes including stereolithography (SLA) and vat photopolymerisation are generally considered less anisotropic than extrusion or PBF methods. Yet even in SLA, build orientation modulates surface roughness and subsurface stress state, and parts produced in an upright orientation show measurably lower elongation at break than those built flat, reflecting the residual influence of inter-layer cure boundaries [213]. Collectively, the anisotropy in AM parts is therefore a systems-level phenomenon coupling process parameters (laser power, scan speed, layer thickness, raster angle), the resulting microstructure (grain morphology, crystallographic texture, molecular diffusion depth), and the external loading direction. Mitigating it requires deliberate control at each of these levels.

Although mechanical anisotropy is most pronounced in material extrusion owing to limited polymer chain diffusion across deposited filaments, other polymer additive manufacturing technologies also exhibit process-dependent anisotropic behavior arising from fundamentally different mechanisms. A comparative understanding of these mechanisms is essential for establishing a universal process–structure–property framework across polymer AM [8,26].

In vat photopolymerization processes, including stereolithography (SLA), digital light processing (DLP), and liquid crystal display (LCD) printing, anisotropy originates primarily from layer-wise photopolymerization rather than filament interfaces. Variations in curing depth, light penetration, crosslink density, and post-curing efficiency generate subtle differences in network structure between successive layers [72,214,215]. These differences become particularly significant in thick components or highly filled photopolymer resins, where optical attenuation and incomplete interlayer conversion may reduce interfacial strength. Residual stresses generated by polymerization shrinkage further contribute to anisotropic deformation and dimensional inaccuracies [102,216]. Compared with FFF, however, vat-photopolymerized components generally exhibit lower mechanical anisotropy because adjacent layers undergo partial chemical bonding during photopolymerization, producing more continuous polymer networks [217,218].

Several material and process optimization strategies have been developed to minimize anisotropy in vat photopolymerization. These include optimization of exposure energy and layer thickness, visible-light photoinitiator systems with improved penetration depth, grayscale or variable-exposure curing strategies, optimized post-curing protocols, and low-shrinkage resin formulations incorporating flexible oligomers or dynamic covalent networks [102,215,219]. Collectively, these approaches improve conversion homogeneity, reduce residual stresses, and strengthen interlayer bonding, resulting in more isotropic mechanical performance [217,220].

Polymer powder bed fusion, particularly selective laser sintering (SLS), exhibits a different anisotropy mechanism governed primarily by powder consolidation and thermal history. During selective laser exposure, incomplete particle coalescence, localized variations in melt-pool temperature, and non-uniform cooling produce directional differences in crystallinity, porosity, and neck formation between neighboring particles [45,221]. Build orientation also influences heat accumulation and cooling rates, leading to anisotropic tensile strength and fracture behavior, especially along the build direction where interlayer fusion is less complete than in the scanning plane [222,223].

Mitigation strategies for polymer powder bed fusion therefore focus on improving thermal uniformity throughout the build. Optimized laser scanning strategies, elevated and uniformly controlled powder-bed temperatures, improved particle size distribution and powder flowability, reduced powder aging, and controlled cooling cycles promote more homogeneous particle coalescence while minimizing residual stresses and crystallization-induced warpage [104,224]. Post-processing treatments such as thermal annealing and hot isostatic consolidation, where applicable, further improve density and reduce microstructural heterogeneity, thereby enhancing isotropic mechanical performance [225,226].

Taken together, the origins of anisotropy differ substantially among polymer AM technologies. Material extrusion is dominated by molecular diffusion across deposited filaments, vat photopolymerization by curing kinetics and crosslink-density gradients, and powder bed fusion by thermal history and particle coalescence [26,227]. Consequently, effective anisotropy mitigation requires process-specific optimization rather than universal parameter adjustment, highlighting the importance of tailoring material design and processing conditions simultaneously to achieve reliable structural performance [15,228].

### 5.2. Layer-by-Layer Defects

The sequential deposition inherent to AM creates numerous opportunities for defect formation that are absent or rare in conventional manufacturing [229]. In metal AM, three families of volumetric defects dominate: lack-of-fusion (LoF) pores, keyhole pores, and gas-entrapped spherical pores. Each arises through a distinct physical mechanism and carries different implications for structural integrity.

Lack-of-fusion defects form when the energy input is insufficient to fully remelt and bond adjacent tracks or successive layers. The resulting voids are irregular in shape, can be on the order of the powder particle size, and are concentrated at the boundaries between melt tracks [230]. They are highly detrimental to fatigue performance because their sharp edges act as potent stress concentrators and crack initiation sites, with fatigue life reductions of one to two orders of magnitude compared with fully dense material reported in the literature [231]. At the opposite energy extreme, keyhole porosity develops when excessive laser power or low scan velocity causes localised vaporisation and the formation of a deep, vapour-filled cavity. Synchrotron X-ray imaging studies have revealed that keyhole collapse generates bubbles that undergo rapid growth through pressure equilibration followed by partial shrinkage driven by metal-vapour condensation; concurrent hydrogen diffusion into the bubble stabilises the residual void against complete closure [232]. Pore formation in the keyhole regime has been shown to be governed by five interdependent factors: vapour condensation, liquid vortex, recoil pressure, surface tension, and keyhole morphology, all of which are set by the laser–material interaction [233].

A recently identified and previously overlooked defect category in LPBF is shrinkage porosity—analogous to the classic casting defect—which forms at the microstructural scale as metal contracts during solidification when liquid backfill pathways become blocked by the advancing solid front [207]. Additionally, disturbances in the shielding gas flow above the powder bed disrupt convective heat transfer in the melt pool, generating patterned keyhole porosity even when laser parameters nominally fall within the stable processing window, highlighting that machine-level variables contribute independently to defect populations [234]. The study by Zhai et al. [235] investigates the bending performance and failure mechanisms of additively manufactured lattice sandwich structures, demonstrating that double-layer SSDCPLC configurations exhibit significantly improved load-carrying capacity and energy absorption compared to conventional designs.

The authors of [236] investigate the additive manufacturing of symmetric corrugated hierarchical honeycombs (SCHHs) using photopolymer-based 3D printing, integrating experimental testing and finite element simulations to evaluate their mechanical performance. The authors demonstrate that incorporating cylindrical support structures and nano-SiO_2_ fillers significantly enhances compressive strength, energy absorption, and stiffness, with optimal performance achieved at ~7 wt.% SiO_2_ loading. Additionally, the work highlights the sensitivity of mechanical properties to filler dispersion, showing that excessive nanoparticle content leads to agglomeration and consequent performance degradation. These findings provide valuable insights into the design and material optimization of architected cellular structures fabricated via additive manufacturing.

Beyond volumetric pores, residual stress is a pervasive layer-level defect that profoundly affects part quality. Repeated rapid heating and cooling cycles introduce steep thermal gradients and constrained thermal contraction, generating tensile residual stresses that can exceed 10 MPa in polymer systems and are even larger in metals [58,237,238]. These stresses promote delamination—the debonding of adjacent layers—particularly at free edges where the combination of peel and shear loading is most severe. The plane-strain stress state in the core of a solidified FDM deposit has been shown analytically to be nearly equibiaxial and tensile, implying that any pre-existing crack is prone to propagation, while the interface between the first and second layer experiences the greatest combined shear and tension, making it the preferential site for delamination [58]. In fibre-reinforced polymer composites produced by extrusion deposition AM, specimens with weak interfacial strength exhibited interlaminar delamination failure under bending loads, directly translating poor interlayer bond quality into macroscopic mechanical degradation [239].

In summary, the defect landscape in AM is multi-dimensional: energy density governs the transition between LoF and keyhole regimes, process atmosphere and gas flow add stochastic variation, and thermal history determines the residual stress that drives interlayer cracking. Characterisation techniques including X-ray computed tomography and synchrotron imaging have been indispensable in quantifying defect populations and elucidating formation mechanisms, providing the physical basis needed to design targeted mitigation strategies.

### 5.3. Post-Processing: Annealing and Curing

Because as-built AM parts invariably carry heterogeneous microstructures, residual stresses, and—in the case of photopolymers—incomplete conversion, post-processing is routinely required to bring properties to the level demanded by structural applications. The two most widely employed strategies, thermal annealing and UV/thermal curing, operate through fundamentally different mechanisms yet share the common objective of homogenising the material state introduced during layer-by-layer fabrication.

**Figure 8:**
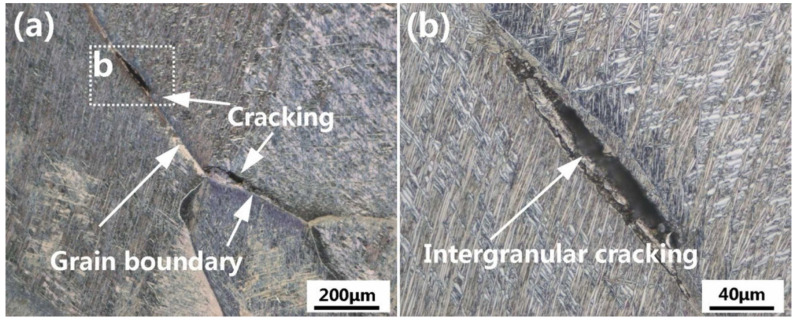
Subsurface micrographs of the specimen annealed at 1020 °C following room-temperature tensile testing: (**a**) low-magnification view and (**b**) high-magnification view [240]. (Figure is available under Open Access).

For metal AM, post-processing heat treatment must contend with microstructural heterogeneity, internal defects, and residual stresses that collectively distinguish AM metals from their conventionally manufactured counterparts in ways that make direct application of established heat treatment schedules unreliable [240]. Stress relief annealing below the recrystallisation temperature reduces residual stresses while preserving the fine as-built grain structure, whereas higher-temperature solution treatments and subsequent ageing cycles dissolve segregation, redistribute precipitates, and restore more isotropic properties. In wire-arc directed energy deposition (DED) of the near-α titanium alloy Ti–6Al–2Zr–1Mo–1V, a single post-deposition annealing treatment was found to partially randomise the strong build-direction texture and reduce the difference in tensile properties between vertical and horizontal orientations, demonstrating that annealing can partially recover isotropy in addition to relieving stress [241]. In Inconel 718 produced by LPBF, post-processing (homogenisation followed by hot isostatic pressing) reorganised the as-built columnar grains with strong {002} texture into equiaxed grains of approximately 150 µm with near-random orientation, largely eliminating the texture-driven anisotropy [242]. Figure 8 presents the subsurface microstructure of the Ti–6Al–2Zr–1Mo–1V alloy annealed at 1020 °C after tensile testing. The figure reveals intergranular crack initiation and propagation along prior β-grain boundaries, caused by dislocation pile-up within coarse α colonies and continuous grain-boundary α layers. These features explain the reduced tensile strength and ductility observed after supertransus annealing.

Hot isostatic pressing (HIP) represents the most comprehensive post-treatment for metal AM components, simultaneously addressing porosity, residual stress, and microstructural heterogeneity [243]. During HIP, parts are subjected to high temperatures and isostatic gas pressure (typically argon or nitrogen), causing pores to close by creep and diffusion bonding; because AM-produced pores are homogeneously distributed and the surface is gas-tight, encapsulation-free HIP is feasible, simplifying the process. The resulting densification is homogeneous, so minimal geometric distortion occurs [244,245]. High-resolution X-ray tomography studies confirm that HIP is highly effective at closing most typical porosity distributions, though highly interconnected near-surface pores represent exceptions; a post-HIP annealing step has been shown to cause a novel ‘blistering’ effect in some alloys, cautioning against uncritical sequential treatment [244]. The fatigue improvement achievable through HIP can reach two orders of magnitude relative to as-built parts, making it indispensable for aerospace and biomedical applications where cyclic loading governs the design life [246].

For polymer AM, thermal annealing acts through two complementary mechanisms: molecular chain diffusion across interlayer interfaces and, in semi-crystalline matrices, enhanced crystallisation. In FDM-printed ABS reinforced with recycled carbon fibre, annealing improved tensile and flexural strength by up to 12.6% and 42.3%, respectively, by narrowing inter-bead voids and reducing fibre–matrix gaps [247]. Post-print annealing of PLA above its glass transition temperature increases crystallinity, reduces void content, and lowers water adsorption, thereby mitigating environmental degradation in aggressive service environments [248]. Liquid crystalline polymers benefit particularly from annealing: the treatment drives molecular alignment and interlayer diffusion simultaneously, with the annealing time governing the kinetics of viscous rearrangement at the interface and setting the ultimate flexural performance [249].

For photopolymer-based processes, UV post-curing is a mandatory step because parts exit the printer in a partially polymerised ‘green’ state containing unreacted monomers and oligomers. Post-curing with flood UV irradiation breaks residual double bonds in the acrylic backbone, extends polymer chains, and creates additional cross-links between chains, substantially improving elastic modulus, tensile strength, and hardness [250]. However, the improvement follows a non-linear relationship with curing time: an optimal window exists beyond which additional irradiation causes over-curing and may embrittle the network by introducing excessive cross-link density or thermal degradation [251]. The incorporation of nanofillers such as graphene nanoplatelets into SLA resins has been shown to improve elastic modulus by up to 104% and hardness by 85% through enhanced load transfer and energy dissipation, and these improvements are complementary to rather than replaced by UV or thermal post-curing [252].

In conclusion, the processing–structure–property relationships in AM are tightly coupled across all length scales. Anisotropy originates in the directional microstructure and weak interlayer bonds imposed by the layer-by-layer process; defects ranging from LoF pores to keyhole cavities and residual stresses arise from the complex thermal history; and post-processing—whether thermal annealing, HIP, or UV curing—provides the essential means to recover properties toward or beyond the levels of conventionally manufactured counterparts. A complete understanding of these linkages is a prerequisite for reliable deployment of AM parts in safety-critical applications.

## 6. Challenges and Limitations

### 6.1. Limited Material Palette

Despite the rapid expansion of polymer additive manufacturing (AM), the range of materials that can be reliably processed remains considerably narrower than that available to conventional manufacturing routes such as injection moulding or compression moulding. The layer-by-layer deposition principle imposes strict rheological and thermal requirements on feedstocks: materials must flow predictably at elevated temperatures, adhere to previously deposited layers, and solidify without excessive warpage or shrinkage. These constraints effectively exclude a large fraction of the industrially relevant polymer space. In fused deposition modelling (FDM), processable materials are largely confined to commodity thermoplastics such as PLA, ABS, and PETG, while high-performance engineering polymers such as PEEK or PEI demand specialised hardware operating at nozzle temperatures exceeding 400 °C, placing them beyond the reach of most production-scale systems [253].

Elastomers present a particularly acute case of material-palette restriction. Although thermoplastic polyurethane (TPU) has gained traction in FDM-based AM, fully crosslinked thermoset elastomers—which offer superior chemical resistance, thermal stability, and mechanical damping—remain exceedingly difficult to process by standard extrusion-based routes because their network structure prevents re-melting [254]. The situation is broadly analogous in vat photopolymerisation (SLA/DLP), where feedstocks are restricted to UV-curable resins that form thermoset networks upon irradiation. While these resins provide fine resolution and smooth surfaces, their thermoset character limits both the achievable property range and the prospects for downstream recycling [10].

Selective laser sintering (SLS), though less restricted than FDM, is dominated in commercial practice by polyamide 12 (PA12) powder, with only a handful of alternative thermoplastic powders—among them PA11, TPU, and polypropylene—meeting the stringent flowability, coalescence, and thermal stability criteria required for consistent sintering [255]. More broadly, the incorporation of conductive fillers (carbon black, carbon nanotubes, graphene) into polymer matrices to impart electrical functionality introduces further processability challenges: excessive filler loading can cause nozzle clogging, reduce interlayer adhesion, and degrade bulk mechanical properties, constraining the achievable multi-functionality of printed parts [31].

### 6.2. Mechanical Anisotropy

Mechanical anisotropy—the direction-dependent variation in strength, stiffness, and fracture resistance—is an intrinsic consequence of the layer-by-layer build strategy and represents one of the most significant barriers to the deployment of polymer AM parts in load-bearing applications. In FDM, the root cause is the poor diffusion and chain entanglement across adjacent filament interfaces: when a new layer is deposited, the underlying surface has already cooled below the glass-transition temperature, curtailing the molecular interdiffusion required for strong weld formation [256].

The result is that FFF/FDM components routinely exhibit their weakest mechanical response along the build (Z) direction, where interlayer bonds are the primary load-carrying elements. Digital image correlation studies on ABS specimens have quantitatively confirmed that stress concentration and damage initiation preferentially occur at interlayer interfaces when loading is applied perpendicular to the print plane [257]. This build-direction weakness is not merely a material property but is sensitive to a complex interplay of processing parameters—layer thickness, raster angle, infill pattern, nozzle temperature, and print speed—making it difficult to predict and control in engineering practice [258].

High-performance polymers such as PEEK are particularly susceptible: the inherently weak interlayer bonding in FDM processing of PEEK severely limits mechanical strength and hinders widespread application in structural contexts, even when otherwise excellent in-plane properties are achieved [259]. Post-processing strategies such as laser-assisted re-heating, hot-rolling of deposited layers, and annealing have demonstrated partial recovery of isotropic behaviour, but add cost and complexity to the production workflow [260]. The anisotropy problem is compounded in composite FFF feedstocks: while continuous or short-fibre reinforcement can substantially raise in-plane tensile strength, the fibre alignment imposed by the extrusion process tends to amplify rather than mitigate directional mechanical differences [261].

### 6.3. Scale-Up and Industrialisation

The translation of polymer AM from a rapid-prototyping tool to a high-throughput manufacturing technology faces fundamental economic and technical obstacles. The layer-by-layer deposition mode is inherently sequential, meaning that build time scales approximately with part volume rather than benefiting from the economies of scale that parallel processes such as injection moulding enjoy at high volumes. Large-format polymer AM is therefore constrained by a persistent tension between surface quality and production speed: increasing deposition rate to improve throughput degrades surface finish and often worsens interlayer bonding, while reducing layer height for quality improvements proportionally extends cycle times [255].

A further bottleneck arises from the largely manual nature of pre- and post-processing steps. Support removal, surface finishing, and part inspection frequently cannot be automated without significant investment, inflating per-part labour costs and limiting throughput [262,263]. Process repeatability and part-to-part consistency—prerequisites for industrial certification—remain challenging because even small environmental variations (ambient temperature, humidity, and filament moisture content) affect final part quality. Widespread adoption of AM by safety-critical industries is consequently slowed by the absence of standardised qualification procedures and material datasheets that match the maturity of conventional manufacturing standards [264].

Size scalability presents a distinct set of engineering challenges. Conventional AM systems are typically limited to build volumes at the sub-metre scale, whereas applications in aerospace, marine, and civil construction demand structures of several metres. Large-scale 3D printing must overcome problems of thermal management across extended build envelopes, where differential cooling causes residual stresses and geometric distortion that are amplified relative to small-scale prints [265]. Efforts to extend polymer AM to metre- and beyond-metre scales—through pellet extrusion, robotic deposition, and segmented assembly strategies—have demonstrated technical feasibility but have yet to achieve the cost-per-part parity with established manufacturing routes needed for broad industrial adoption [266].

### 6.4. Recycling and Sustainability Issues

Polymer AM is often characterised as an inherently sustainable technology on account of its near-net-shape fabrication and reduced subtractive waste. However, closer scrutiny reveals a more complex picture. The recyclability of AM polymers is strongly constrained by material chemistry. Thermoset resins—central to SLA and DLP processes—form irreversible covalent networks upon curing, and consequently cannot be re-melted or reprocessed by conventional mechanical recycling routes [267]. Life-cycle assessment studies confirm that thermoset resins in photopolymerisation AM currently represent a largely unresolved end-of-life challenge, with most printed parts entering landfill or incineration streams at end of use [268].

**Figure 9:**
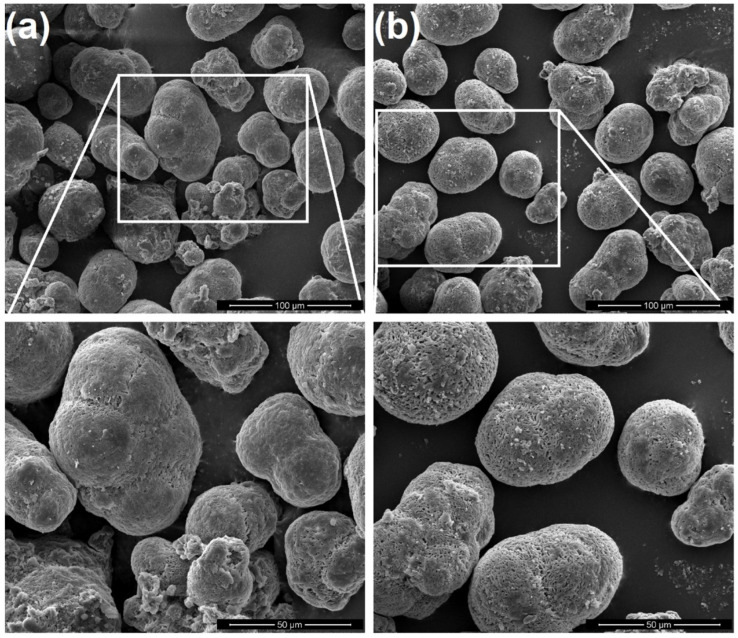
Secondary electron SEM images showing (**a**) virgin PA12 powder and (**b**) powder recovered after 12 reuse cycles [270]. (Figure is available under Open Access).

Thermoplastic feedstocks used in FDM/FFF are in principle recyclable, but repeated thermal processing degrades molecular weight, crystallinity, and rheological consistency, progressively compromising part quality. Investigations into recycled PLA and ABS filaments demonstrate that while mechanical properties can remain competitive for single recycling cycles, multiple recycles induce embrittlement and colour change that limit viable reuse to a small number of generations [269]. In SLS, where a substantial fraction of powder—in some cases 85–95%—remains unsintered after each build, the unsintered material can in principle be blended with fresh powder for subsequent runs; however, repeated thermal exposure degrades powder flowability and part quality, effectively creating a secondary waste stream that must be managed [270,271,272]. Figure 9 compares the surface morphology of virgin PA12 powder and PA12 powder after 12 consecutive SLS printing cycles, where the reused powder was subjected to milling, filtering (200 μm mesh), and homogenization after each cycle, without the addition of virgin powder. The SEM images reveal that repeated thermal exposure during SLS processing leads to noticeable morphological changes, with the aged powder exhibiting a rougher particle surface and an apparent increase in particle porosity compared to the virgin material. These changes are attributed to powder aging caused by prolonged exposure to temperatures close to the melting point (~180 °C) and repeated heating–cooling cycles. The observed increase in powder porosity is particularly relevant because it may contribute to the higher porosity later detected in printed parts, indicating a progressive degradation of powder quality with repeated reuse.

The shift from mono-material to composite AM feedstocks—driven by the need for superior mechanical or functional performance—introduces a further sustainability tension. Multi-material and fibre-reinforced printed parts are inherently more difficult to separate and recycle than their neat-polymer counterparts, potentially worsening the environmental footprint of AM relative to the single-material baseline [273]. Developing a genuine circular economy for polymer AM therefore requires not only improvements to recyclability at the material level—for instance through the design of reversible thermosets based on dynamic covalent bonds—but also life-cycle thinking embedded into materials selection, process design, and end-of-life logistics from the outset [274]. The energy intensity of AM processes themselves also warrants attention: FDM machines operate continuously at elevated temperatures and, depending on the electricity mix, can carry a non-negligible carbon footprint even before accounting for material production and transport [275].

Beyond improving material reuse, recent research has increasingly shifted toward designing polymer systems that inherently support circular manufacturing principles. Rather than treating recycling as an end-of-life operation, current material development aims to enable repair, remanufacturing, and repeated reuse through rational polymer chemistry and product design [276,277].

One important strategy is the adoption of design-for-disassembly in multi-material additive manufacturing. Although multi-material printing enables highly integrated structures with spatially tailored mechanical, electrical, and thermal properties, it also complicates recycling because permanently bonded dissimilar materials are difficult to separate after service [278,279]. Consequently, researchers have proposed separable interface designs, reversible joining strategies, mechanically detachable architectures, and modular component design to facilitate selective material recovery without sacrificing functional integration. Such approaches support closed-loop manufacturing by allowing valuable functional materials to be recovered, refurbished, and reintroduced into subsequent manufacturing cycles [280,281].

A second major development is the emergence of dynamic covalent polymer networks, including vitrimers and covalent adaptable networks (CANs), as recyclable feedstocks for additive manufacturing. Unlike conventional thermosetting photopolymers that form permanently crosslinked networks, dynamic covalent polymers contain reversible chemical bonds capable of exchange reactions under external stimuli such as heat or light [282,283,284]. These reversible reactions permit stress relaxation, self-healing, reshaping, welding, and multiple recycling cycles while maintaining structural integrity. Dynamic chemistries based on transesterification, imine exchange, disulfide exchange, boronic ester exchange, and Diels–Alder reactions have been successfully incorporated into photocurable resins and extrusion-compatible thermoplastic systems, providing an attractive route toward sustainable functional materials for vat photopolymerization and material extrusion. Recent studies further highlight their potential to reduce thermoset waste while extending component service life through repair and reprocessing [88,285,286,287].

In parallel, considerable effort has been devoted to developing recyclable functional polymer feedstocks without compromising advanced performance. Recycled PLA, PETG, polyamide, and polypropylene have been reformulated with stabilizers, chain extenders, and functional nanofillers to recover mechanical properties after multiple processing cycles [288,289,290]. Bio-derived polymers, chemically recyclable polyesters, and vitrimer-based composites have likewise demonstrated promising mechanical performance together with significantly improved material circularity. Machine-learning-assisted optimization of recycled feedstock composition and processing parameters is further emerging as an effective strategy for maintaining printability and property consistency despite inevitable variations in recycled material quality [147,291].

Taken together, these developments illustrate that sustainability in polymer additive manufacturing is evolving from simple feedstock reuse toward a holistic circular economy framework encompassing recyclable polymer chemistry, design-for-disassembly, repairable multifunctional materials, and digitally enabled material lifecycle management. Future additive manufacturing systems will therefore increasingly integrate sustainable material design with intelligent manufacturing strategies to minimize waste while preserving the high performance required for advanced engineering applications [292,293].

### 6.5. Summary of Key Limitations in Polymer AM and Nanocomposites in a Table

For the reader’s convenience, the main limitations in Polymer AM and Nanocomposites are summarized in Table 1.

## 7. Emerging Trends and Future Perspectives

Polymer-based additive manufacturing (AM) is undergoing a period of rapid transformation, driven by convergent advances in materials science, computational intelligence, and manufacturing engineering. The frontiers now extend well beyond geometric complexity, encompassing adaptive functionality, intelligent process control, and digitally designed material systems. This chapter examines four defining directions: multi-material and hybrid printing, 4D printing with time-dependent functionality, integration with machine learning (ML), and digital materials design and simulation.

### 7.1. Multi-Material and Hybrid Printing

The ability to deposit multiple materials in a single fabrication cycle has fundamentally expanded the design space for polymer AM. Advances in multi-material additive manufacturing (MMAM) now make it feasible to fabricate complex hybrid structures with high spatial resolution by combining dissimilar materials, enabling properties that are unattainable in monolithic parts [294]. In the realm of polymer–metal hybridity, laser-based powder bed fusion (LPBF) surfaces have been engineered with gecko-like micro-pillar anchoring features to maximise adhesion at polymer–metal interfaces, achieving competitive quasi-static mechanical strengths [295]. A complementary strategy employs dual-nozzle fused deposition modelling (FDM) printers that simultaneously deposit conductive and insulating polymers, enabling the fabrication of integrated electronic circuits without post-assembly steps [31].

High-performance thermoplastics are also being leveraged in multi-material architectures. Polyetherimide (PEI)-based systems produced by multi-material FDM demonstrate robust, lightweight structures with high-temperature stability, positioning them as viable candidates for aerospace components where weight reduction and thermal resilience are critical [296]. At the interface of electronics and structural design, laser-activated selective electroless plating on FDM-printed substrates has been demonstrated for customised 3D electronics, yielding functional metallic circuitry directly on printed polymer geometries [297]. More recently, cold-spray-assisted electroless deposition has enabled selective surface metallisation of 3D-printed polymers, broadening the toolkit for hybrid fabrication without high-temperature processing [298].

### 7.2. 4D Printing: Time-Dependent Functionality

Four-dimensional (4D) printing extends AM by incorporating the dimension of time, yielding structures that change shape, properties, or functionality in response to external stimuli after fabrication. Formally defined as “3D printing + time,” the approach relies on stimuli-responsive programmable materials—including shape-memory polymers (SMPs), hydrogels, and liquid-crystal elastomers—to achieve self-assembly, multi-functionality, and self-repair without additional assembly steps [19].

Shape-memory thermomorphs represent one of the most intensively studied material classes for 4D printing. These substances encompass shape-memory alloys, shape-memory polymers, shape-memory gels, and shape-memory liquid-crystal elastomers, which can change physical properties precisely in response to thermal stimulation, enabling dynamic and adaptive structures across the fourth dimension of time [122]. Thermo-induced shape-memory polymers (TSMPs) in particular have seen a surge of research attention, with recent comprehensive reviews documenting their switching mechanisms, constitutive models, and application trajectories across biomedicine, robotics, and aerospace [299]. Digital light processing has been employed to fabricate multi-SMP constructs within a single material precursor, achieving spatio-temporal tunability of shape-memory transition temperature, rubbery modulus, and maximum elongation of up to 250%, all within a printing time of 30 s [300].

Stimuli-responsive hydrogels broaden the repertoire further, responding to temperature, pH, moisture, magnetism, redox conditions, and light. A recent review framing these materials within a multidimensional comparative system—connecting stimulus-response chemistry with fabrication technique and end-use domain—highlights engineering guidelines for overcoming practical barriers such as mechanical fragility, slow actuation kinetics, and limited environmental stability [301]. In the biomedical domain, 4D-printed shape-memory polymers and programmable hydrogels are enabling a paradigm shift, with self-healing hydrogels already demonstrating promise in cartilage repair by autonomously restoring structural integrity in response to microfractures [121]. Suspension bath printing of temperature-responsive granular hydrogel inks has further advanced the ability to fabricate shape-morphing soft materials with well-controlled actuation profiles for soft robotics and implantable devices [302]. Looking forward, the convergence of 4D printing with regenerative medicine represents one of the most clinically impactful frontiers, offering patient-specific therapies and next-generation adaptive implants [303,304].

### 7.3. Integration with Machine Learning

Machine learning is reshaping every layer of polymer AM—from material formulation and printing parameter selection to real-time quality assurance and predictive property modelling. A comprehensive analysis spanning polymers, metals, ceramics, and carbon-based materials confirms that ML-driven approaches are now central to process optimisation, quality assurance, and predictive modelling across AM platforms [305].

Supervised and reinforcement learning have proven particularly effective for parameter optimisation. Multi-objective optimisation of drop-on-demand printing, for example, has been achieved via fully connected neural networks that simultaneously minimise droplet diameter and maximise droplet speed, using inputs such as applied voltage, viscosity, surface tension, and nozzle diameter [306]. For polymer composites more broadly, ML techniques—including supervised, unsupervised, and deep learning—offer an efficient alternative to resource-intensive physical testing by analysing large datasets to uncover structure–property relationships and predict behaviour under complex processing conditions [307].

Real-time defect detection represents another high-impact application domain. Vision-based deep learning systems have been deployed for in situ FDM monitoring, with transformer-based architectures such as the Data-efficient Image Transformer (DeiT), combined with contrast-limited adaptive histogram equalisation pre-processing, achieving average defect detection accuracy of 97.6% for common defects including stringing and spaghetti-like layer failures [308]. Convolutional neural networks (CNNs) integrated with reinforcement learning frameworks pave the way for adaptive, self-improving quality control systems capable of autonomous correction within Industry 4.0 manufacturing environments [309]. Lightweight deployment variants based on improved YOLOv8 architectures have demonstrated real-time detection with a mean average precision (mAP50) of 97.5%, an 18.1% increase in frames-per-second throughput, and a 32.9% reduction in computational cost [310].

**Figure 10:**
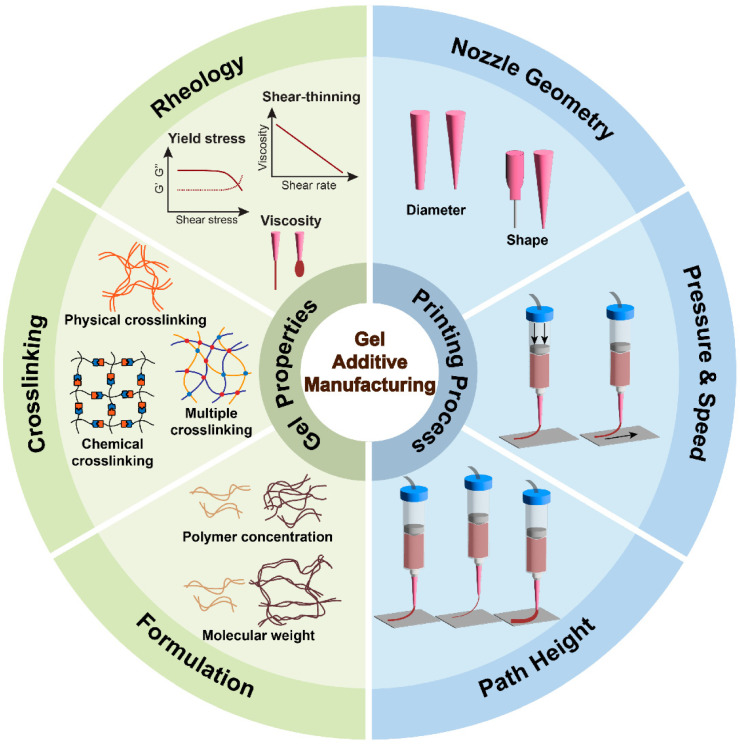
Interaction of material properties and printing conditions in gel-based additive manufacturing [312]. (This figure is available under Open Access).

At the material design level, ML-based parameter optimisation has become a critical component of intelligent polymer design, encompassing both process-level control during polymer forming and molecular-level property prediction [311]. Gel-based AM has emerged as a particularly fertile ground for these methods, where ML accelerates material discovery and provides predictive process optimisation across print fidelity and rheological properties [312]. Figure 10 provides a schematic overview of the key relationships governing gel-based additive manufacturing. It highlights how critical material properties—including viscosity, shear-thinning behavior, viscoelasticity, yield stress, and crosslinking mechanisms—interact with process parameters such as nozzle diameter, extrusion pressure, printing speed, and path height. Together, these interconnected factors determine filament continuity, extrusion behavior, shape fidelity, and the structural integrity of printed constructs. The figure serves as a conceptual framework for understanding gel printability and establishes the basis for subsequent machine-learning-driven optimization of material formulations and printing conditions.

Recent advances in artificial intelligence (AI) and machine learning (ML) are transforming polymer additive manufacturing from an empirical trial-and-error process into a data-driven intelligent manufacturing paradigm. Rather than serving isolated optimization tasks, ML algorithms increasingly support every stage of the additive manufacturing lifecycle, forming an integrated closed-loop framework that links material design, process control, quality assurance, and structural performance prediction [9,313,314].

#### 7.3.1. Molecular and Material Design

The first stage focuses on material discovery and formulation optimization. Machine learning models trained on experimental databases and molecular descriptors enable rapid prediction of polymer properties, including glass transition temperature, viscosity, crystallization behavior, tensile strength, electrical conductivity, and printability [315,316]. Bayesian optimization, Gaussian process regression, graph neural networks, and deep learning algorithms are increasingly employed to identify optimal monomer compositions, nanofiller concentrations, photoinitiator systems, and processing additives while substantially reducing experimental effort. In functional polymer development, inverse design approaches further allow researchers to specify target properties before computationally identifying candidate material formulations [229,317].

#### 7.3.2. Intelligent Process Parameter Optimization

Once suitable materials are identified, AI supports optimization of printing parameters across different additive manufacturing technologies. Machine learning algorithms establish complex nonlinear relationships between material properties, process parameters, and final part quality that are difficult to capture using conventional analytical models [318,319,320]. Parameters including nozzle temperature, laser power, scan speed, exposure energy, raster angle, layer thickness, and build orientation can be optimized simultaneously to maximize dimensional accuracy, mechanical performance, and production efficiency [321,322]. Reinforcement learning and adaptive optimization algorithms increasingly enable closed-loop process regulation, allowing printing parameters to be adjusted automatically in response to changing process conditions [323,324].

#### 7.3.3. In Situ Process Monitoring and Defect Detection

The third stage integrates real-time sensing with artificial intelligence for online quality assurance. High-speed cameras, infrared thermography, acoustic emission sensors, force monitoring, optical coherence tomography, and melt-pool imaging generate large volumes of process data that can be analyzed using convolutional neural networks, vision transformers, and anomaly detection algorithms [322,325,326]. These approaches enable early identification of defects including porosity, delamination, insufficient fusion, warpage, dimensional deviations, and surface irregularities before print completion. Increasingly, defect prediction is coupled with closed-loop feedback systems capable of automatically modifying process parameters to mitigate defect formation during printing rather than after fabrication [327,328].

#### 7.3.4. Long-Term Structural Performance Prediction and Digital Lifecycle Management

Beyond manufacturing itself, machine learning is expanding toward prediction of component performance throughout the service lifecycle. Physics-informed neural networks, digital twins, and hybrid finite element–machine learning models are increasingly used to estimate fatigue life, creep deformation, environmental degradation, residual stress evolution, thermal aging, and structural reliability under complex service conditions [329,330,331]. By continuously integrating manufacturing data, inspection records, and operational monitoring, digital twin frameworks provide predictive maintenance capabilities and enable data-driven decisions regarding repair, refurbishment, or component replacement. Such approaches are particularly valuable for aerospace, biomedical, and energy applications, where long-term reliability is essential [332,333].

Collectively, these developments establish a full-lifecycle intelligent manufacturing framework in which machine learning supports every stage of polymer additive manufacturing, from molecular-scale material design to end-of-life performance assessment. Future intelligent AM systems are expected to integrate computational materials design, autonomous process optimization, real-time defect correction, and digital lifecycle management within unified cyber-physical manufacturing platforms, significantly accelerating the development of next-generation functional polymer components [334,335,336].

### 7.4. Digital Materials Design and Simulation

The ability to design materials computationally—before committing any material to a printer—is becoming indispensable in polymer AM. Computational methods, spanning quantum chemical simulations, coarse-grained force fields, and data-driven ML, can pre-screen and select optimal polymer formulations tailored to specific printing technologies and target performance criteria, drastically shortening the development cycle [337]. Machine-learning-assisted molecular design follows a structured pipeline encompassing database construction, structural representation and feature engineering, development of property prediction models, virtual screening of candidates, and experimental validation—enabling structure–property relationships to be extracted and molecular architectures optimised with unprecedented speed [338].

At the structural scale, multiscale computational modelling has been applied to large-format AM of carbon-fibre-reinforced polymer composites, integrating mean-field homogenisation with finite element analysis to characterise anisotropic mechanical behaviour and predict warpage arising from thermal gradients between deposited layers—a critical consideration for aerospace and automotive tooling [34]. Multiscale simulation frameworks are also being applied to continuously fibre-reinforced 3D-printed composites to predict macroscale mechanical properties from molecular-level processes, reducing the time and cost associated with exhaustive experimental characterisation [339].

Digital twin technology represents the most integrative expression of simulation-driven manufacturing. Digital twins—computer-generated models that mirror the real-time behaviour of physical AM systems—have been extensively reviewed as mechanisms for quality control, process optimisation, and cost reduction in polymer AM, with the DT market projected to expand from $3.8 billion in 2019 to $35.8 billion by 2025 [340]. Simulation-in-the-loop frameworks, in which finite element models run concurrently with live printing, have enabled real-time structural validation and dynamic process adjustment [341]. Hybrid digital twins that couple finite element thermal history computation with ML-based residual stress prediction have been shown to proactively modify infill patterns during printing, substantially reducing warpage and eliminating delamination in carbon-fibre-reinforced polymer parts [342].

A proposed AI-assisted whole life cycle framework integrates material genetic design, intelligent manufacturing, and end-of-life recycling considerations, offering a roadmap for embedding computational design not only at the process level but across the entire material lifespan of polymer AM products [36]. Together, these developments point towards a future where polymer AM is governed not by empirical trial-and-error but by closed-loop digital intelligence—a paradigm in which material properties, manufacturing outcomes, and part performance are predicted, validated, and optimised before a single layer is deposited.

## 8. Conclusions

Additive manufacturing has evolved from a rapid prototyping technology into a versatile platform for fabricating functional materials with tailored mechanical, electrical, thermal, and biological properties. This review has demonstrated that advances in functional polymers—including conductive, stimuli-responsive, elastomeric, high-performance, bio-based, and nanocomposite systems—have substantially expanded the capabilities of additive manufacturing across biomedical, aerospace, energy, electronics, and soft robotic applications. Furthermore, the discussion of polymer- and metal-based additive manufacturing highlights that, despite differences in material behavior and processing physics, both material families are governed by the same fundamental processing–structure–property relationships that ultimately determine component performance and reliability.

Despite remarkable progress, several scientific challenges remain unresolved and continue to limit broader industrial adoption. These challenges can be grouped into four principal categories. First, materials-related limitations include the relatively narrow portfolio of printable engineering polymers and alloys, insufficient understanding of structure–property relationships in multifunctional materials, limited compatibility of functional fillers with existing printing processes, and the absence of standardized material qualification protocols. Second, process-related challenges arise from the inherently layer-wise nature of additive manufacturing, resulting in anisotropic properties, residual stresses, porosity, interlayer defects, dimensional inaccuracies, and process instability. While these issues manifest differently in polymer extrusion, vat photopolymerization, powder bed fusion, and metal laser processing, they all originate from incomplete control of heat transfer, phase transformations, and interfacial bonding during fabrication. Third, characterization and modeling remain significant bottlenecks, as predictive models capable of simultaneously describing processing, microstructure evolution, and long-term functional performance are still limited, particularly for multifunctional composites and multi-material architectures. Finally, industrial implementation is constrained by limited process repeatability, insufficient in situ quality assurance, high feedstock costs, lengthy qualification procedures, and the lack of harmonized standards for certification of safety-critical components.

Addressing these challenges requires research strategies tailored to different material families and manufacturing modalities. For polymer-based additive manufacturing, future efforts should prioritize the development of high-performance recyclable polymers, sustainable bio-derived feedstocks, multifunctional nanocomposites with improved filler dispersion, and advanced resin and filament formulations specifically engineered for individual printing processes. Greater emphasis should also be placed on understanding molecular diffusion, crystallization kinetics, rheological behavior, and degradation mechanisms to improve both printability and long-term reliability. For metal additive manufacturing, research priorities should focus on controlling solidification pathways, minimizing residual stresses and defect formation, expanding printable alloy systems, and developing process-specific post-processing strategies that enhance microstructural homogeneity while reducing manufacturing costs. Across both polymer and metal systems, the development of robust multi-material fabrication strategies and functionally graded materials represents a critical opportunity for creating components with spatially tailored properties and integrated multifunctionality.

Over the next five years, the field is expected to transition from empirical process optimization toward predictive, data-driven materials engineering. Artificial intelligence, machine learning, digital twins, high-throughput materials discovery, and physics-informed computational models are likely to become integral components of additive manufacturing workflows, enabling simultaneous optimization of material composition, process parameters, and component performance. Equally important will be the integration of real-time sensing, in situ monitoring, and closed-loop process control to reduce variability, improve reproducibility, and accelerate qualification for industrial production.

From an industrial perspective, successful translation of functional additive manufacturing will depend on moving beyond demonstrations of material performance toward reliable, scalable manufacturing ecosystems. This will require standardized testing methodologies, qualification frameworks for emerging material systems, lifecycle and sustainability assessments, improved feedstock recyclability, automated quality assurance, and economically viable manufacturing routes capable of consistent large-scale production. Close collaboration between materials scientists, manufacturing engineers, computational researchers, equipment manufacturers, and end users will be essential for establishing robust digital manufacturing platforms that integrate materials design, process optimization, structural characterization, and performance prediction.

Overall, the future of additive manufacturing lies not only in the discovery of new functional materials but also in the convergence of advanced materials science, intelligent process control, computational design, and industrial digitalization. Such an integrated approach will enable the rational design and reliable production of next-generation multifunctional components with predictable performance, thereby accelerating the widespread adoption of additive manufacturing in high-value engineering and biomedical applications.

## Figures and Tables

**Table 1:** Challenges and Limitations regarding Polymer AM and Nanocomposites.

Process/Domain	Materials & System	Applications	Key Limitations/Challenges	Refs.
FDM/FFF of High-Performance Polymers	PEEK, PEKK, PEI (ULTEM 9085/1010); high-temp thermoplastics	Aerospace, defense, space structures	Residual stresses, incomplete interlayer diffusion, porosity → reduced fatigue life and reliability	[58,146,147,148]
Post-processing (Vacuum Annealing)	Annealing above Tg, below Tm; improves crystallinity	Aerospace-grade parts, space manufacturing	Requires tight thermal control; risk of distortion; adds processing step and cost	[149,150,151,152,153,154]
Thermo-mechanical consolidation (IR + rollers)	Continuous CF/PEEK composites; elevated temp + pressure	Structural aerospace laminates	Requires specialized hardware; process complexity; integration challenges	[155,156,157,158,159]
Bio-based polymers (PLA)	PLA; composites with β-TCP, bioglass (↑ strength up to ~80%)	Biomedical scaffolds, fixation devices	Brittleness; low thermal stability; void formation increases with layer thickness (0.2–0.4 mm)	[167,168,169]
Bio-based polymers (PHAs)	PHA blends; elongation >2000% vs. PLA ~29%	Flexible biodegradable parts, biomedical	Low global production share (~0.2%); cost and scalability limitations	[171,172,173]
Nanoparticle-reinforced thermoplastics	Si_3_N_4_ (4 wt.%), TiN (6 wt.%), AlN (2 wt.%) → strength ↑30–50%	Structural lightweight components	Narrow optimal loading window; viscosity increase affects printability	[176,177,178]
Oxide nanofillers in polymers	SiO_2_ in PLA (≤1 wt.% optimal); hollow SiO_2_ > 5 wt.% → degradation	Antimicrobial, thermal management	Agglomeration beyond threshold → mechanical decline; non-linear behavior	[179,180]
Metal nanoparticles (Cu in PLA/TPU)	Cu^0^/Cu^+^/Cu^2+^ species	Antibacterial materials	Thermal processing alters surface chemistry → reduced functionality	[181]
Short fiber composites (FDM)	SCF/PP: tensile ↑35%, bending ↑40%	Structural parts, conductive components	Strong anisotropy due to fiber alignment along print path	[184,185]
Continuous fiber composites	CF/PEEK; ILSS ↑172% with treatment	High-performance aerospace	Poor fiber–matrix bonding → delamination; requires post-processing	[186,187]
Carbon nanomaterials (CNTs, graphene)	CNT loading limited (SLA: ~0.05 wt.%); GNP up to 5 wt.% → strength ↑67%	Electronics, conductive structures	UV absorption limits photopolymerization; dispersion challenges	[188,189,190,191,192,193]
MXene nanocomposites	Ti_3_C_2_T_x_ (~0.5 wt.%) → strength ↑32–43%	Sensors, EMI shielding, electronics	Rheology tuning required; emerging material → scalability issues	[195,196,197,198,199]
Extrusion AM (nanocomposites)	Viscosity increases with filler loading	General AM parts	Nozzle clogging; melt rheology limits printability; nozzle wear from abrasive fillers	[200,201]
Vat photopolymerization (SLA/DLP)	Resin viscosity limit: ~0.1–10 Pa·s	High-resolution printing	UV attenuation (e.g., CNTs reduce penetration depth by 90% at 0.25 wt.%)	[95,193]
Filler loading thresholds	SiO_2_/PLA (1 wt.%), TiN/ABS (6 wt.%), Si_3_N_4_/ABS (4 wt.%)	Multi-functional composites	Non-monotonic behavior; agglomeration beyond threshold reduces performance	[176,180]
Dispersion & functionalization	Sonication, compatibilizers, surface treatment	All nanocomposites	Trade-off: better dispersion vs. degradation of intrinsic nanoparticle properties	[204]
DIW (Direct Ink Writing)	Requires shear-thinning inks; nozzle size 100–800 µm	Flexible electronics, sensors	Strict rheological window (yield stress, G′, viscosity); difficult optimization	[196,205]
General AM (all processes)	Layer-by-layer fabrication	All structural applications	Mechanical anisotropy due to interlayer bonding and directional microstructure	[206]

## Data Availability

No new data were created or analyzed in this study. Data sharing is not applicable to this article.

## References

[1] Koltsaki M., Mavri M. (2024). A Comprehensive Overview of Additive Manufacturing Processes Through a Time-Based Classification Model. 3D Print. Addit. Manuf..

[2] ben Said L., Ayadi B., Alharbi S., Dammak F. (2025). Recent Advances in Additive Manufacturing: A Review of Current Developments and Future Directions. Machines.

[3] Chaudhary R., Fabbri P., Leoni E., Mazzanti F., Akbari R., Antonini C. (2023). Additive manufacturing by digital light processing: A review. Prog. Addit. Manuf..

[4] Rosnitschek T., Baumann T., Orgeldinger C., Alber-Laukant B., Tremmel S. (2023). Manufacturing Constraints in Topology Optimization for the Direct Manufacturing of Extrusion-Based Additively Manufactured Parts. Designs.

[5] Häfele T., Schneberger J.H., Buchholz S., Vielhaber M., Griebsch J. (2023). The impact of geometric complexity on manufacturing process efficiency of Selective Laser Sintering. Procedia CIRP.

[6] Fidan I., Huseynov O., Ali M.A., Alkunte S., Rajeshirke M., Gupta A., Hasanov S., Tantawi K., Yasa E., Yilmaz O. (2023). Recent Inventions in Additive Manufacturing: Holistic Review. Inventions.

[7] Sonkamble V., Phafat N. (2023). A current review on electron beam assisted additive manufacturing technology: Recent trends and advances in materials design. Discov. Mech. Eng..

[8] Vicente C.M.S., Sardinha M., Reis L., Ribeiro A., Leite M. (2023). Large-format additive manufacturing of polymer extrusion-based deposition systems: Review and applications. Prog. Addit. Manuf..

[9] Nasrin T., Pourkamali-Anaraki F., Peterson A.M. (2024). Application of machine learning in polymer additive manufacturing: A review. J. Polym. Sci..

[10] Chyr G., DeSimone J.M. (2022). Review of high-performance sustainable polymers in additive manufacturing. Green Chem..

[11] Muehlenfeld C., Duffy P., Yang F., Zermeño Pérez D., El-Saleh F., Durig T. (2024). Excipients in Pharmaceutical Additive Manufacturing: A Comprehensive Exploration of Polymeric Material Selection for Enhanced 3D Printing. Pharmaceutics.

[12] Golhin A.P., Tonello R., Frisvad J.R., Grammatikos S., Strandlie A. (2023). Surface roughness of as-printed polymers: A comprehensive review. Int. J. Adv. Manuf. Technol..

[13] Alghamdi S.S., John S., Choudhury N.R., Dutta N.K. (2021). Additive manufacturing of polymer materials: Progress, promise and challenges. Polymers.

[14] González-Henríquez C.M., Sarabia-Vallejos M.A., Rodriguez-Hernandez J. (2019). Polymers for additive manufacturing and 4D-printing: Materials, methodologies, and biomedical applications. Prog. Polym. Sci..

[15] Yu K., Dunn M.L., Qi H.J., Maute K. (2025). Recent advances in design optimization and additive manufacturing of composites: From enhanced mechanical properties to innovative functionalities. npj Adv. Manuf..

[16] Shen W., Zhang P., Li W., Qin H. (2026). Additive manufacturing for functional design: A review of capabilities, strategies and applications. Rapid Prototyp. J..

[17] Iervolino F., Suriano R., Cavallaro M., Castoldi L., Levi M. (2025). Additively Manufactured Polymers for Electronic Components. Appl. Sci..

[18] Shabaniverki S., Juárez J.J. (2021). Directed assembly of particles for additive manufacturing of particle-polymer composites. Micromachines.

[19] Valvez S., Reis P.N.B., Susmel L., Berto F. (2021). Fused filament fabrication-4d-printed shape memory polymers: A review. Polymers.

[20] Chu H., Yang W., Sun L., Cai S., Yang R., Liang W., Yu H., Liu L. (2020). 4D printing: A review on recent progresses. Micromachines.

[21] Chanraksmey L., Nie B., Zhao W., Thummavichai K., Chen Y., Chen B. (2026). Additive manufacturing of functional high-performance polymers for energy storage, conversion, and insulation systems. Smart Mater. Manuf..

[22] Sarabia-Vallejos M.A., Rodríguez-Umanzor F.E., González-Henríquez C.M., Rodríguez-Hernández J. (2022). Innovation in Additive Manufacturing Using Polymers: A Survey on the Technological and Material Developments. Polymers.

[23] Das A., Gilmer E.L., Biria S., Bortner M.J. (2021). Importance of Polymer Rheology on Material Extrusion Additive Manufacturing: Correlating Process Physics to Print Properties. ACS Appl. Polym. Mater..

[24] Wang Z., Wang L., Tang F., Chen J. (2025). Multi-material additive manufacturing via fused deposition modeling 3D printing: A systematic review on the material feeding mechanism. Proc. Inst. Mech. Eng. Part E J. Process Mech. Eng..

[25] Gao X., Qi S., Kuang X., Su Y., Li J., Wang D. (2021). Fused filament fabrication of polymer materials: A review of interlayer bond. Addit. Manuf..

[26] Zohdi N., Yang R.C. (2021). Material anisotropy in additively manufactured polymers and polymer composites: A review. Polymers.

[27] Wu H., Fahy W.P., Kim S., Kim H., Zhao N., Pilato L., Kafi A., Bateman S., Koo J.H. (2020). Recent developments in polymers/polymer nanocomposites for additive manufacturing. Prog. Mater. Sci..

[28] Adam H.M. (2026). Emerging advantages and transformative applications of nanocomposites in additive manufacturing: A literature review. Next Res..

[29] Ingram M., Campbell E., Molotnikov A., Feih S., Zhong Y.L. (2024). Recent advances in multifunctional polymer/2D nanocomposite development for fused filament fabrication and direct ink writing of electrically and thermally conductive components. Adv. Nanocompos..

[30] García-Collado A., Blanco J.M., Gupta M.K., Dorado-Vicente R. (2022). Advances in polymers based Multi-Material Additive-Manufacturing Techniques: State-of-art review on properties and applications. Addit. Manuf..

[31] Pradeep Raja C., Vigneshwaren S., Parrthipan B.K., Babu S., Kathik Babu N.B., Mensah R.A. (2025). Additive manufacturing of polymers and composites for sustainable engineering applications. Front. Chem. Eng..

[32] de Sousa Alves B.A., Kontziampasis D., Soliman A.H. (2024). The Quest for the Holy Grail of 3D Printing: A Critical Review of Recycling in Polymer Powder Bed Fusion Additive Manufacturing. Polymers.

[33] Wang Z., Yang W., Liu Q., Zhao Y., Liu P., Wu D., Banu M., Chen L. (2022). Data-driven modeling of process, structure and property in additive manufacturing: A review and future directions. J. Manuf. Process..

[34] Castelló-Pedrero P., García-Gascón C., García-Manrique J.A. (2024). Multiscale numerical modeling of large-format additive manufacturing processes using carbon fiber reinforced polymer for digital twin applications. Int. J. Mater. Form..

[35] Castro J., Nóbrega J.M., Costa R. (2024). Computational Framework to Model the Selective Laser Sintering Process. Materials.

[36] Liu Z., Han J., Wang S., Li J., Yi H., Long T. (2025). Research Progress and Future Perspectives of 3D Printing Polymer-Based Materials Whole Life Cycle Frameworks: Material Genetic Design–Intelligent Manufacturing–Recycling. Adv. Eng. Mater..

[37] Nath S.D., Nilufar S. (2020). An overview of additive manufacturing of polymers and associated composites. Polymers.

[38] Brighenti R., Cosma M.P., Marsavina L., Spagnoli A., Terzano M. (2021). Laser-based additively manufactured polymers: A review on processes and mechanical models. J. Mater. Sci..

[39] Khan I., Barsoum I., Abas M., al Rashid A., Koç M., Tariq M. (2024). A review of extrusion-based additive manufacturing of multi-materials-based polymeric laminated structures. Compos. Struct..

[40] Ligon S.C., Liska R., Stampfl J., Gurr M., Mülhaupt R. (2017). Polymers for 3D Printing and Customized Additive Manufacturing. Chem. Rev..

[41] Regis J.E., Renteria A., Hall S.E., Hassan M.S., Marquez C., Lin Y. (2021). Recent trends and innovation in additive manufacturing of soft functional materials. Materials.

[42] Rajeshirke M., Fidan I., Naikwadi V., Alkunte S., Gupta A., Mohammadizadeh M. (2025). Material extrusion–based multi-material 3D printing: A holistic review of recent advances. Int. J. Adv. Manuf. Technol..

[43] Minetola P., Calignano F., Galati M. (2020). Comparing geometric tolerance capabilities of additive manufacturing systems for polymers. Addit. Manuf..

[44] Jafferson J.M., Chatterjee D. (2021). A review on polymeric materials in additive manufacturing. Mater. Today Proc..

[45] Chatham C.A., Long T.E., Williams C.B. (2019). A review of the process physics and material screening methods for polymer powder bed fusion additive manufacturing. Prog. Polym. Sci..

[46] Wiese M., Thiede S., Herrmann C. (2020). Rapid manufacturing of automotive polymer series parts: A systematic review of processes, materials and challenges. Addit. Manuf..

[47] Yeganeh M., Shahryari Z., Shoushtari M.T., Eskandari M. (2025). Discontinuities in the laser powder bed fusion alloys: A review. J. Mater. Res. Technol..

[48] Das A., Bryant J.S., Williams C.B., Bortner M.J. (2023). Melt-Based Additive Manufacturing of Polyolefins Using Material Extrusion and Powder Bed Fusion. Polym. Rev..

[49] Vock S., Klöden B., Kirchner A., Weißgärber T., Kieback B. (2019). Powders for powder bed fusion: A review. Prog. Addit. Manuf..

[50] Chatham C.A., Bortner M.J., Johnson B.N., Long T.E., Williams C.B. (2021). Predicting mechanical property plateau in laser polymer powder bed fusion additive manufacturing via the critical coalescence ratio. Mater. Des..

[51] Lupone F., Padovano E., Casamento F., Badini C. (2022). Process phenomena and material properties in selective laser sintering of polymers: A review. Materials.

[52] Bräuer G., Sachsenhofer K., Lang R.W. (2021). Material and process engineering aspects to improve the quality of the bonding layer in a laser-assisted fused filament fabrication process. Addit. Manuf..

[53] Coogan T.J., Kazmer D.O. (2020). Prediction of interlayer strength in material extrusion additive manufacturing. Addit. Manuf..

[54] McIlroy C., Olmsted P.D. (2017). Disentanglement effects on welding behaviour of polymer melts during the fused-filament-fabrication method for additive manufacturing. Polymer.

[55] Mahmoud Y., Lyu J., Akhavan J., Xu K., Manoochehri S. (2023). Thermal history based prediction of interlayer bond strength in parts manufactured by material extrusion additive manufacturing. Int. J. Adv. Manuf. Technol..

[56] Vaes D., Coppens M., Goderis B., Zoetelief W., van Puyvelde P. (2021). The extent of interlayer bond strength during fused filament fabrication of nylon copolymers: An interplay between thermal history and crystalline morphology. Polymers.

[57] Moetazedian A., Allum J., Gleadall A., Silberschmidt V.v. (2023). Bulk-Material Bond Strength Exists in Extrusion Additive Manufacturing for a Wide Range of Temperatures, Speeds, and Layer Times. 3D Print. Addit. Manuf..

[58] Sreejith P., Kannan K., Rajagopal K.R. (2023). A thermodynamic framework for the additive manufacturing of crystallizing polymers. Part I: A theory that accounts for phase change, shrinkage, warpage and residual stress. Int. J. Eng. Sci..

[59] Zerriouh A., Deghiche A., Benayache W., Hashemi T., Bernardi A., Liparoti S., di Lorenzo M.L., Cavallo D. (2025). Warpage in Material Extrusion Additive Manufacturing of Amorphous and Semicrystalline Polymers. Macromol. Mater. Eng..

[60] Spoerk M., Holzer C., Gonzalez-Gutierrez J. (2020). Material extrusion-based additive manufacturing of polypropylene: A review on how to improve dimensional inaccuracy and warpage. J. Appl. Polym. Sci..

[61] Behseresht S., Park Y.H., Love A., Valdez Pastrana O.A. (2024). Application of Numerical Modeling and Finite Element Analysis in Fused Filament Fabrication: A Review. Materials.

[62] Leśniowski J., Stawiarski A., Barski M. (2025). Enhancing the Performance of FFF-Printed Parts: A Review of Reinforcement and Modification Strategies for Thermoplastic Polymers. Materials.

[63] Cuan-Urquizo E., Barocio E., Tejada-Ortigoza V., Pipes R.B., Rodriguez C.A., Roman-Flores A. (2019). Characterization of the mechanical properties of FFF structures and materials: A review on the experimental, computational and theoretical approaches. Materials.

[64] Rau D.A., Bortner M.J., Williams C.B. (2023). A rheology roadmap for evaluating the printability of material extrusion inks. Addit. Manuf..

[65] Schwab A., Levato R., D’Este M., Piluso S., Eglin D., Malda J. (2020). Printability and Shape Fidelity of Bioinks in 3D Bioprinting. Chem. Rev..

[66] Acierno D., Patti A. (2023). Fused Deposition Modelling (FDM) of Thermoplastic-Based Filaments: Process and Rheological Properties—An Overview. Materials.

[67] Kim S.K., Kazmer D.O. (2022). Non-isothermal non-Newtonian three-dimensional flow simulation of fused filament fabrication. Addit. Manuf..

[68] Gilmer E.L., Miller D., Chatham C.A., Zawaski C., Fallon J.J., Pekkanen A., Long T.E., Williams C.B., Bortner M.J. (2018). Model analysis of feedstock behavior in fused filament fabrication: Enabling rapid materials screening. Polymer.

[69] Schuller T., Fanzio P., Galindo-Rosales F.J. (2022). Analysis of the importance of shear-induced elastic stresses in material extrusion. Addit. Manuf..

[70] Behdani B., Senter M., Mason L., Leu M., Park J. (2020). Numerical study on the temperature-dependent viscosity effect on the strand shape in extrusion-based additive manufacturing. J. Manuf. Mater. Process..

[71] Bakrani Balani S., Chabert F., Nassiet V., Cantarel A. (2019). Influence of printing parameters on the stability of deposited beads in fused filament fabrication of poly(lactic) acid. Addit. Manuf..

[72] Pagac M., Hajnys J., Ma Q.P., Jancar L., Jansa J., Stefek P., Mesicek J. (2021). A review of vat photopolymerization technology: Materials, applications, challenges, and future trends of 3D printing. Polymers.

[73] de Camargo I.L., Morais M.M., Fortulan C.A., Branciforti M.C. (2021). A review on the rheological behavior and formulations of ceramic suspensions for vat photopolymerization. Ceram. Int..

[74] Schittecatte L., Geertsen V., Bonamy D., Nguyen T., Guenoun P. (2023). From resin formulation and process parameters to the final mechanical properties of 3D printed acrylate materials. MRS Commun..

[75] Caplins B.W., Kolibaba T.J., Arp U., Miller C.C., Zong Y., Poster D.L., Higgins C.I., Killgore J.P. (2024). Influence of spectral bandwidth on the working curve in vat photopolymerization. Addit. Manuf..

[76] Kolibaba T.J., Killgore J.P., Caplins B.W., Wendland R.J., Arp U., Miller C.C., Zong Y., Higgins C.I., Sharp J.L., Broce S. (2025). Results of an interlaboratory study on the working curve in vat photopolymerization II: Towards a standardized method. Addit. Manuf..

[77] Dumur F. (2023). The future of visible light photoinitiators of polymerization for photocrosslinking applications. Eur. Polym. J..

[78] Shao J., Huang Y., Fan Q. (2014). Visible light initiating systems for photopolymerization: Status, development and challenges. Polym. Chem..

[79] Enayati-Gerdroodbar A., Khayati A., Ahmadi M., Pourabbas B., Ali Aboudzadeh M., Salami-Kalajahi M. (2024). An overview on potential of novel photoinitiators for vat photopolymerization-based 3D/4D printing formulations. Eur. Polym. J..

[80] Ligon S.C., Husár B., Wutzel H., Holman R., Liska R. (2014). Strategies to reduce oxygen inhibition in photoinduced polymerization. Chem. Rev..

[81] Shi S., Croutxé-Barghorn C., Allonas X. (2017). Photoinitiating systems for cationic photopolymerization: Ongoing push toward long wavelengths and low light intensities. Prog. Polym. Sci..

[82] Fang H., Guymon C.A. (2022). Recent advances to decrease shrinkage stress and enhance mechanical properties in free radical polymerization: A review. Polym. Int..

[83] Marx P., Wiesbrock F. (2021). Expanding monomers as anti-shrinkage additives. Polymers.

[84] Maurya S.D., Kurmvanshi S.K., Mohanty S., Nayak S.K. (2018). A Review on Acrylate-Terminated Urethane Oligomers and Polymers: Synthesis and Applications. Polym. Plast. Technol. Eng..

[85] Stansbury J.W. (2012). Dimethacrylate network formation and polymer property evolution as determined by the selection of monomers and curing conditions. Dent. Mater..

[86] Halloran J.W. (2016). Ceramic Stereolithography: Additive Manufacturing for Ceramics by Photopolymerization. Annu. Rev. Mater. Res..

[87] Lei Z., Chen H., Huang S., Wayment L.J., Xu Q., Zhang W. (2024). New Advances in Covalent Network Polymers via Dynamic Covalent Chemistry. Chem. Rev..

[88] Winne J.M., Leibler L., du Prez F.E. (2019). Dynamic covalent chemistry in polymer networks: A mechanistic perspective. Polym. Chem..

[89] Pruksawan S., Chong Y.T., Zen W., Loh T.J.E., Wang F.K. (2024). Sustainable Vat Photopolymerization-Based 3D-Printing through Dynamic Covalent Network Photopolymers. Chem. Asian J..

[90] Subedi S., Liu S., Wang W., Naser Shovon S.M.A., Chen X., Ware H.O.T. (2024). Multi-material vat photopolymerization 3D printing: A review of mechanisms and applications. npj Adv. Manuf..

[91] Weng Z., Huang X., Peng S., Zheng L., Wu L. (2023). 3D printing of ultra-high viscosity resin by a linear scan-based vat photopolymerization system. Nat. Commun..

[92] Sameni F., Ozkan B., Zarezadeh H., Karmel S., Engstrøm D.S., Sabet E. (2022). Hot Lithography Vat Photopolymerisation 3D Printing: Vat Temperature vs. Mixture Design. Polymers.

[93] Okoruwa L., Tarak F., Sameni F., Sabet E. (2025). Bridging Experimentation and Computation: OMSP for Advanced Acrylate Characterization and Digital Photoresin Design in Vat Photopolymerization. Polymers.

[94] Shaukat U., Rossegger E., Schlögl S. (2022). A Review of Multi-Material 3D Printing of Functional Materials via Vat Photopolymerization. Polymers.

[95] Shah M., Ullah A., Azher K., Rehman A.U., Juan W., Aktürk N., Tüfekci C.S., Salamci M.U. (2023). Vat photopolymerization-based 3D printing of polymer nanocomposites: Current trends and applications. RSC Adv..

[96] Tehfe M.A., Louradour F., Lalevée J., Fouassier J.P. (2013). Photopolymerization reactions: On the way to a green and sustainable chemistry. Appl. Sci..

[97] Lee T.Y., Guymon C.A., Jönsson E.S., Hoyle C.E. (2004). The effect of monomer structure on oxygen inhibition of (meth)acrylates photopolymerization. Polymer.

[98] Karalekas D., Aggelopoulos A. (2003). Study of shrinkage strains in a stereolithography cured acrylic photopolymer resin. J. Mater. Process. Technol..

[99] Sekmen K., Rehbein T., Johlitz M., Lion A., Constantinescu A. (2023). Curing-dependent thermo-viscoelastic and shrinkage behaviour of photopolymers. Mech. Mater..

[100] Fuh J.Y.H., Chooo Y.S., Lu L., Nee A.Y.C., Wong Y.S., Wang W.L., Miyazawa T., Ho S.H. (1997). Post-cure shrinkage of photo-sensitive material used in laser lithography process. J. Mater. Process. Technol..

[101] Bao Y. (2022). Recent Trends in Advanced Photoinitiators for Vat Photopolymerization 3D Printing. Macromol. Rapid Commun..

[102] Schwartz J.J. (2022). Additive manufacturing: Frameworks for chemical understanding and advancement in vat photopolymerization. MRS Bull..

[103] Zhang F., Zhu L., Li Z., Wang S., Shi J., Tang W., Li N., Yang J. (2021). The recent development of vat photopolymerization: A review. Addit. Manuf..

[104] Zinatlou Ajabshir S., Mohammadkamal H., Zinatlou Ajabshir Z., Barletta D., Caiazzo F., Poletto M. (2026). Polymeric Powders for Powder Bed Fusion: From Chemistry and Powder Characteristics to Process Parameters, Defects and Applications. Polymers.

[105] George A., Bryant J.S., Taylor T., Bortner M.J., Williams C.B., Dadmun M.D. (2025). Impact of polymer molecular weight blends on the powder bed fusion process and the properties of polypropylene printed parts. RSC Appl. Polym..

[106] Chatham C.A. (2025). Crystallization-coalescence relationships in laser powder bed fusion: Moving beyond the “sintering window”. Addit. Manuf..

[107] Sanders B., Cant E., Kelly C.A., Jenkins M. (2024). The Effect of Powder Re-Use on the Coalescence Behaviour and Isothermal Crystallisation Kinetics of Polyamide 12 within Powder Bed Fusion. Polymers.

[108] Teo H.W.B., Chen K., Tran V.T., Du H., Zeng J., Zhou K. (2021). Non-isothermal crystallization behaviour of polyamide 12 analogous to multi-jet fusion additive manufacturing process. Polymer.

[109] Ho I., Bryant J., Chatham C., Williams C. (2025). Process parameter optimization in polymer powder bed fusion of final part properties in polyphenylene sulfide through design of experiments. Prog. Addit. Manuf..

[110] da Conceição M.d.N., Anaya-Mancipe J., Bastos D.C., Pereira P.S.C., Libano E.V.D.G. (2025). Influence of Additional Devices and Polymeric Matrix on In Situ Welding in Material Extrusion: A Review. Processes.

[111] Goh G.D., Yap Y.L., Tan H.K.J., Sing S.L., Goh G.L., Yeong W.Y. (2020). Process–Structure–Properties in Polymer Additive Manufacturing via Material Extrusion: A Review. Crit. Rev. Solid State Mater. Sci..

[112] Yan Y., Han M., Jiang Y., Ng E.L.L., Zhang Y., Owh C., Song Q., Li P., Loh X.J., Chan B.Q.Y. (2024). Electrically Conductive Polymers for Additive Manufacturing. ACS Appl. Mater. Interfaces.

[113] Blachowicz T., Ehrmann G., Ehrmann A. (2023). Recent Developments in Additive Manufacturing of Conductive Polymer Composites. Macromol. Mater. Eng..

[114] Li W., Li Y., Song Z., Wang Y.X., Hu W. (2024). PEDOT-based stretchable optoelectronic materials and devices for bioelectronic interfaces. Chem. Soc. Rev..

[115] Oh B., Baek S., Nam K.S., Sung C., Yang C., Lim Y.S., Ju M.S., Kim S., Kim T.S., Park S.M. (2024). 3D printable and biocompatible PEDOT:PSS-ionic liquid colloids with high conductivity for rapid on-demand fabrication of 3D bioelectronics. Nat. Commun..

[116] Zhang X., Lu C., Zhang Y., Cai Z., He Y., Liang X. (2025). In Situ 3D Printing of Conformal Bioflexible Electronics via Annealing PEDOT:PSS/PVA Composite Bio-Ink. Polymers.

[117] Khan M., Refati M.F.A.D., Arup M.M.R., Islam M.A., Mobarak M.H. (2025). Conductive Polymer-Based Electronics in Additive Manufacturing: Materials, Processing, and Applications. Adv. Polym. Technol..

[118] Zhang P., Zhu B., Du P., Travas-Sejdic J. (2024). Electrochemical and Electrical Biosensors for Wearable and Implantable Electronics Based on Conducting Polymers and Carbon-Based Materials. Chem. Rev..

[119] Dominguez-Alfaro A., Mitoudi-Vagourdi E., Dimov I., Picchio M.L., Lopez-Larrea N., de Lacalle J.L., Tao X., Serrano R.R.M., Gallastegui A., Vassardanis N. (2024). Light-Based 3D Multi-Material Printing of Micro-Structured Bio-Shaped, Conducting and Dry Adhesive Electrodes for Bioelectronics. Adv. Sci..

[120] Wang Z., Zhang Z., Kuang X. (2025). Recent advances in polymer 4D printing: 3D printing techniques, smart material design, and healthcare applications. Smart Mater. Med..

[121] Alanazi B.N., Ahmed H.A., Alharbi N.S., Ebrahim N.A.A., Soliman S.M.A. (2025). Exploring 4D printing of smart materials for regenerative medicine applications. RSC Adv..

[122] Ding A., Tang F., Alsberg E. (2025). The Emerging 4D Printing of Shape-Memory Thermomorphs for Self-Adaptative Biomedical Implants. Adv. Funct. Mater..

[123] Sadraei A., Naghib S.M. (2025). 4D Printing of Physical Stimuli-Responsive Hydrogels for Localized Drug Delivery and Tissue Engineering. Polym. Rev..

[124] Abdullah T., Okay O. (2023). 4D Printing of Body Temperature-Responsive Hydrogels Based on Poly(acrylic acid) with Shape-Memory and Self-Healing Abilities. ACS Appl. Bio Mater..

[125] Song X., Zhang W., Liu H., Zhao L., Chen Q., Tian H. (2022). 3D printing of liquid crystal elastomers-based actuator for an inchworm-inspired crawling soft robot. Front. Robot. AI.

[126] Kotikian A., Watkins A.A., Bordiga G., Spielberg A., Davidson Z.S., Bertoldi K., Lewis J.A. (2024). Liquid Crystal Elastomer Lattices with Thermally Programmable Deformation via Multi-Material 3D Printing. Adv. Mater..

[127] Long G., Deng Y., Zhao W., Zhou G., Broer D.J., Feringa B.L., Chen J. (2024). Photoresponsive Biomimetic Functions by Light-Driven Molecular Motors in Three Dimensionally Printed Liquid Crystal Elastomers. J. Am. Chem. Soc..

[128] Sadraei A., Naghib S.M., Rabiee N. (2025). 4D printing chemical stimuli-responsive hydrogels for tissue engineering and localized drug delivery applications–part 2. Expert Opin. Drug Deliv..

[129] Ni C., Zhang C., Qin Z., Ke Q., Wu B., Zhuoruo X., Sun Y., Fu Q., Chen D., Zheng N. (2025). 4D printing of trigger-free shape-memory hydrogels towards self-adaptive substrates for bioelectronics. Nat. Commun..

[130] Bliah O., Hegde C., Tan J.M.R., Magdassi S. (2025). Fabrication of Soft Robotics by Additive Manufacturing: From Materials to Applications. Chem. Rev..

[131] Zhou L.Y., Gao Q., Fu J.Z., Chen Q.Y., Zhu J.P., Sun Y., He Y. (2019). Multimaterial 3D Printing of Highly Stretchable Silicone Elastomers. ACS Appl. Mater. Interfaces.

[132] Yamagishi K., Karyappa R., Ching T., Hashimoto M. (2024). Direct ink writing of silicone elastomers to fabricate microfluidic devices and soft robots. MRS Commun..

[133] Raj R., Song Q., Juang J. (2025). Multi-Material Additive Manufacturing of Soft Robotic Systems: A Comprehensive Review. Adv. Robot. Res..

[134] Sadasivuni K.K., Ponnamma D., Rajan M., Basheer Ahamed M., Al-Maadeed M.A.S.A. (2019). Polymer Nanocomposites in Biomedical Engineering.

[135] Sharma B., Phan P.T., Davies J., Hoang T.T., Nguyen C.C., Ji A., Zhu K., Nicotra E., Lovell N.H., Do T.N. (2024). Soft Upper-Limb Wearable Robotic Devices: Technology and Applications. Adv. Intell. Syst..

[136] Vidakis N., Petousis M., Spyridaki M., Mountakis N., Dimitriou E., Michailidis N. (2026). Ultra- and high-performance polymers for material extrusion additive manufacturing: Recent advancements, challenges, and optimization perspectives. Mater. Sci. Eng. R Rep..

[137] Rendas P., Figueiredo L., Machado C., Mourão A., Vidal C., Soares B. (2023). Mechanical performance and bioactivation of 3D-printed PEEK for high-performance implant manufacture: A review. Prog. Biomater..

[138] Kennedy S.M., Raghav G.R., Jeen Robert R.B., Manikandaraja G., Selvakumar M. (2024). PEEK-based 3D printing: A paradigm shift in implant revolution for healthcare. Polym.-Plast. Technol. Mater..

[139] Cojocaru V., Frunzaverde D., Miclosina C.O., Marginean G. (2022). The Influence of the Process Parameters on the Mechanical Properties of PLA Specimens Produced by Fused Filament Fabrication—A Review. Polymers.

[140] Sikder P., Challa B.T., Gummadi S.K. (2022). A comprehensive analysis on the processing-structure-property relationships of FDM-based 3-D printed polyetheretherketone (PEEK) structures. Materialia.

[141] Jonckers D., Gundlach C., Hartwig S., Thakur A. (2025). Characterisation of high-performance polymer parts produced by low-pressure additive manufacturing. CEAS Space J..

[142] Yi N., Davies R., Chaplin A., McCutchion P., Ghita O. (2021). Slow and fast crystallising poly aryl ether ketones (PAEKs) in 3D printing: Crystallisation kinetics, morphology, and mechanical properties. Addit. Manuf..

[143] Doyle L., Pérez-Ferrero X., García-Molleja J., Losada R., Romero-Rodríguez P., Fernández-Blázquez J.P. (2024). Fused Filament Fabrication of Slow-Crystallizing Polyaryletherketones: Crystallinity and Mechanical Properties Linked to Processing and Post-Treatment Parameters. Polymers.

[144] Klenosky D.R., Kitt B.R. Thermoplastic additive manufacturing-ultem 9085 for structural applications. Proceedings of the Composites and Advanced Materials Expo, CAMX 2020.

[145] Intel Market Research (2025). Polymer Additive Manufacturing Equipment Market Outlook 2025–2032. https://www.intelmarketresearch.com/polymer-additive-equipment-market-8888.

[146] Kobler E., Birtha J., Marschik C., Straka K., Steinbichler G., Zwicklhuber P., Schlecht S. (2022). A Novel Multi-Region, Multi-Phase, Multi-Component-Mixture Modeling Approach to Predicting the Thermodynamic Behaviour of Thermoplastic Composites during the Consolidation Process. Polymers.

[147] Hoang V.T., Kwon B.S., Sung J.W., Choe H.S., Oh S.W., Lee S.M., Kweon J.H., Nam Y.W. (2023). Postprocessing method-induced mechanical properties of carbon fiber-reinforced thermoplastic composites. J. Thermoplast. Compos. Mater..

[148] van de Werken N., Koirala P., Ghorbani J., Doyle D., Tehrani M. (2021). Investigating the hot isostatic pressing of an additively manufactured continuous carbon fiber reinforced PEEK composite. Addit. Manuf..

[149] Rusakov D., Menner A., Spieckermann F., Wilhelm H., Bismarck A. (2022). Morphology and properties of foamed high crystallinity PEEK prepared by high temperature thermally induced phase separation. J. Appl. Polym. Sci..

[150] Riggins A.W., Dadmun M.D. (2024). Multiple Molecular-Level Processes in the Evolution of Residual Strain in 3D Printed Poly(ether ether ketone). ACS Appl. Polym. Mater..

[151] Colin X. (2022). Humid and Thermal Oxidative Ageing of Radiation Cured Polymers—A Brief Overview. Front. Chem..

[152] Ahmad J., Niasar M.G. (2025). Aging Behavior of PEEK, PTFE, and PI Insulation Materials Under Thermal Oxidative and Humid Conditions for Aerospace Applications. J. Appl. Polym. Sci..

[153] Maloney A., Manaf E., Gately N., Major I., Devine D.M. (2025). Annealing 3D printed PEKK: Investigating the impact of annealing on the mechanical, physical, and thermal properties in additively manufactured PEKK. Mater. Des..

[154] Vindokurov I., Pirogova Y., Tashkinov M., Silberschmidt V.V. (2022). Effect of Heat Treatment on Elastic Properties and Fracture Toughness of Fused Filament Fabricated PEEK for Biomedical Applications. Polymers.

[155] Liang J., Li J., Li H., Li Y., Zhang S., Zhao X. (2025). Temperature field analysis for automated placement with thermoplastic composites based on the line-focused infrared heater. J. Thermoplast. Compos. Mater..

[156] Venkatesan C., Zulkifli F., Silva A. (2023). Effects of processing parameters of infrared-based automated fiber placement on mechanical performance of carbon fiber-reinforced thermoplastic composite. Compos. Struct..

[157] Chen Y., Qu X., Fan C., Song W., Zheng J.H., He L. (2025). Influence of Process Parameters in Infrared Radiation AFP In Situ Consolidation on the Mechanical Properties of Thermoplastic Composites. Polym. Compos..

[158] Yu X., Song W., Zheng J.H., Shan Z., Chen Y., Fan C., Sun L., Tian A. (2025). Improving interlaminar shear strength of continuous carbon fiber reinforced PEEK via laser directed energy deposition: Experimental study and physically based modelling. Compos. Part B Eng..

[159] Wu D., Miao Q., Dai Z., Niu F., Ma G. (2022). Effect of voids and crystallinity on the interlaminar shear strength of in-situ manufactured CF/PEEK laminates using repass treatment. Compos. Sci. Technol..

[160] Ericson J., Widerker D., Stibbe E., Elgarisi M., Katzman Y., Luria O., Gommed K., Razin A., Hari A.A., Gabay I. (2026). Modeling the thermal behavior of photopolymers for in-space fabrication. npj Microgravity.

[161] Liu T., Zhang M., Kang Y., Tian X., Ding J., Li D. (2023). Material extrusion 3D printing of polyether ether ketone in vacuum environment: Heat dissipation mechanism and performance. Addit. Manuf..

[162] Oromiehie E., das Chakladar N., Rajan G., Prusty B.G. (2019). Online monitoring and prediction of thermo-mechanics of AFP based thermoplastic composites. Sensors.

[163] Heathman N., Koirala P., Yap T., Emami A., Tehrani M. (2023). In situ consolidation of carbon fiber PAEK via laser-assisted automated fiber placement. Compos. Part B Eng..

[164] Denkena B., Schmidt C., Timmermann M., Friedel A. (2022). An optical-flow-based monitoring method for measuring translational motion in infrared-thermographic images of AFP processes. Prod. Eng..

[165] Jha S., Akula B., Enyioma H., Novak M., Amin V., Liang H. (2024). Biodegradable Biobased Polymers: A Review of the State of the Art, Challenges, and Future Directions. Polymers.

[166] Arif Z.U., Khalid M.Y., Noroozi R., Sadeghianmaryan A., Jalalvand M., Hossain M. (2022). Recent advances in 3D-printed polylactide and polycaprolactone-based biomaterials for tissue engineering applications. Int. J. Biol. Macromol..

[167] Sultan S., Thomas N., Varghese M., Dalvi Y., Joy S., Hall S., Mathew A.P. (2022). The Design of 3D-Printed Polylactic Acid–Bioglass Composite Scaffold: A Potential Implant Material for Bone Tissue Engineering. Molecules.

[168] bin Firoz A., Rybakov V., Fetisova A.A., Shlapakova L.E., Pariy I.O., Toropkov N., Lozhkomoev A.S., Mukhortova Y.R., Sharonova A.A., Wagner D.V. (2025). 3D-printed biodegradable composite poly(lactic acid)-based scaffolds with a shape memory effect for bone tissue engineering. Adv. Compos. Hybrid Mater..

[169] Gajjar T., Yang R., Ye L., Zhang Y.X. (2025). Effects of key process parameters on tensile properties and interlayer bonding behavior of 3D printed PLA using fused filament fabrication. Prog. Addit. Manuf..

[170] Park H., He H., Yan X., Liu X., Scrutton N.S., Chen G.Q. (2024). PHA is not just a bioplastic!. Biotechnol. Adv..

[171] Ďurfina M., Babaei N., Vanovčanová Z., Feranc J., Horváth V., Vašková I., Kruželák J., Tomanová K., Plavec R. (2025). Bio-Based Polyhydroxyalkanoate (PHA) Blends for 3D Printing: Rheological, Mechanical, Biocompatibility, and Biodegradation Properties. Polymers.

[172] Kanabenja W., Passornraprasit N., Aumnate C., Osswald T.A., Aht-Ong D., Potiyaraj P. (2024). Enhancing 3D printability of polyhydroxybutyrate (PHB) and poly(3-hydroxybutyrate-co-3-hydroxy valerate) (PHBV) based blends through melt extrusion based-3D printing. Addit. Manuf..

[173] Börner T., Zinn M. (2024). Key challenges in the advancement and industrialization of biobased and biodegradable plastics: A value chain overarching perspective. Front. Bioeng. Biotechnol..

[174] Nath D., Misra M., Al-Daoud F., Mohanty A.K. (2025). Studies on poly(butylene succinate) and poly(butylene succinate-co-adipate)-based biodegradable plastics for sustainable flexible packaging and agricultural applications: A comprehensive review. RSC Sustain..

[175] Rahman M.M., Islam S., Mubasshira, Islam M.S., Ahammad R., Islam M.A., Hasib M.A., Rahman M.S., Moshwan R., Ehsan M.M. (2026). Polymer Composites in Additive Manufacturing: Current Technologies, Applications, and Emerging Trends. Polymers.

[176] Petousis M., Michailidis N., Papadakis V.M., Korlos A., Mountakis N., Argyros A., Dimitriou E., Charou C., Moutsopoulou A., Vidakis N. (2023). Optimizing the Rheological and Thermomechanical Response of Acrylonitrile Butadiene Styrene/Silicon Nitride Nanocomposites in Material Extrusion Additive Manufacturing. Nanomaterials.

[177] Vidakis N., Mangelis P., Petousis M., Mountakis N., Papadakis V., Moutsopoulou A., Tsikritzis D. (2023). Mechanical Reinforcement of ABS with Optimized Nano Titanium Nitride Content for Material Extrusion 3D Printing. Nanomaterials.

[178] Vidakis N., Petousis M., Mangelis P., Maravelakis E., Mountakis N., Papadakis V., Neonaki M., Thomadaki G. (2022). Thermomechanical Response of Polycarbonate/Aluminum Nitride Nanocomposites in Material Extrusion Additive Manufacturing. Materials.

[179] Vidakis N., Petousis M., Velidakis E., Mountakis N., Tzounis L., Liebscher M., Grammatikos S.A. (2021). Enhanced mechanical, thermal and antimicrobial properties of additively manufactured polylactic acid with optimized nano silica content. Nanomaterials.

[180] Asiri J.M. (2026). 3D-Printed Hollow Silica Nanocomposites: A Synergistic Approach to Thermal Insulation and Mechanical Reinforcement in Advanced Polymer Systems. J. Mater. Eng. Perform..

[181] Pinho A.C., Morais P.V., Pereira M.F., Piedade A.P. (2025). Changes in the Antibacterial Performance of Polymer-Based Nanocomposites Induced by Additive Manufacturing Processing. Polymers.

[182] Patiño-Almanza R., García-Méndez R.F., Rivera-Armenta J.L., Strachota A., Almendarez-Camarillo A. (2024). 3D printing polypropylene composites reinforced with functionalized halloysite: Balance between stiffness and impact resistance. Polym. Compos..

[183] Bagatella S., Castoldi L., Cavallaro M., Gariboldi E., Suriano R., Levi M. (2025). 3D-Printable Polymer Composites with Unmodified Boron Nitride for Thermal Management in Flexible Electronics. J. Appl. Polym. Sci..

[184] Zhan X., Su K., Tuo X., Gong Y. (2024). Mechanical, Thermal, and Electrical Properties on 3D Printed Short Carbon Fiber Reinforced Polypropylene Composites. ACS Appl. Polym. Mater..

[185] Estefani A., Távara L. (2024). Numerical multiscale analysis of 3D printed short fiber composites parts: Filament anisotropy and toolpath effects. Eng. Rep..

[186] Lyu Y., Li A., Wu J., Koutsos V., Wang C., Brádaigh C.M.Ó., Yang D. (2025). Enhanced mechanical performance of 3D printed continuous carbon fibre reinforced polyphenylene sulphide composites through dopamine treatment and post-processing compression. Compos. Part A Appl. Sci. Manuf..

[187] Qayyum J.A., Li A., Wu J., Lyu Y., Koutsos V., Zhang H., Radacsi N., Yang D. (2025). On the printability and inter-layer adhesion in 3D printing of continuous carbon fibre reinforced PEEK composite using tape and filament. Compos. Part A Appl. Sci. Manuf..

[188] Kumar V., Singh V., Bansal A., Bharat N., Setia G. (2026). Sustainable 3D-Printed PLA/Graphene Nanocomposites with Enhanced Mechanical and Thermal Performance. J. Inorg. Organomet. Polym. Mater..

[189] Harishbabu S., Alrasheedi N.H., Louhichi B., Sahu S.K., Ma Q. (2025). Optimization and Prediction of Mechanical Properties of Additively Manufactured PLA/GNP Composites via Response Surface Methodology and Machine Learning Models. Polymers.

[190] Spinelli G., Kotsilkova R., Ivanov E., Petrova-Doycheva I., Menseidov D., Georgiev V., di Maio R., Silvestre C. (2020). Effects of filament extrusion, 3D printing and hot-pressing on electrical and tensile properties of poly(Lactic) acid composites filled with carbon nanotubes and graphene. Nanomaterials.

[191] Raimondo M., Aliberti F., Calabrese E., Pantani R., Verde T.R., Sorrentino A., Guadagno L. (2025). 3D Printed Materials with Electrical Properties Tailored for Thermal Management. Macromol. Symp..

[192] Thirugnanasambandam A., Amith S.C., Santhosh S., Mukil T., Harsha R., Dharun N.K. (2025). Mechanical Performance of PLA-MWCNT Nanocomposite Developed by Ecofriendly Hybrid Filament Processing Method for Fused Deposition Modeling Applications. Polym. Compos..

[193] Ahmad K.H., Mohamad Z., Khan Z.I., Habib M. (2025). Tailoring UV Penetration Depth in Photopolymer Nanocomposites: Advancing SLA 3D Printing Performance with Nanofillers. Polymers.

[194] Tilve-Martinez D., Poulin P. (2025). Vat Photopolymerization 3D Printing of Conductive Nanocomposites. Acc. Mater. Res..

[195] Li Y., Kankala R.K., Chen A.Z., Wang S.B. (2022). 3D Printing of Ultrathin MXene toward Tough and Thermally Resistant Nanocomposites. Nanomaterials.

[196] Xu L., Chen W., Li Q., Gupta R., Bagchi B., Lovat L.B., Tiwari M.K. (2025). Printing Nacre-Mimetic MXene-Based E-Textile Devices for Sensing and Breathing-Pattern Recognition Using Machine Learning. Adv. Funct. Mater..

[197] Lee S.J., Choi E., Choi J.Y., Kim C.L. (2026). High-performance wearable pressure sensors based on biocompatible 3D-printed PU sponges with MXene coating. Compos. Part A Appl. Sci. Manuf..

[198] Gebrekrstos A., Muzata T.S., Elias A., Ray S.S. (2025). Tailoring the Properties of 2D Nanomaterial-Polymer Composites for Electromagnetic Interference Shielding and Energy Storage by 3D Printing—A Review. Adv. Eng. Mater..

[199] Xue T., Yang Y., Yu D., Wali Q., Wang Z., Cao X., Fan W., Liu T. (2023). 3D Printed Integrated Gradient-Conductive MXene/CNT/Polyimide Aerogel Frames for Electromagnetic Interference Shielding with Ultra-Low Reflection. Nano-Micro Lett..

[200] Ozdemir B., Hernández-del-Valle M., Gaunt M., Schenk C., Echevarría-Pastrana L., Fernández-Blázquez J.P., Wang D.Y., Haranczyk M. (2024). Toward 3D printability prediction for thermoplastic polymer nanocomposites: Insights from extrusion printing of PLA-based systems. Addit. Manuf..

[201] Clarissa W.H.Y., Chia C.H., Zakaria S., Evyan Y.C.Y. (2022). Recent advancement in 3-D printing: Nanocomposites with added functionality. Prog. Addit. Manuf..

[202] Sevriugina V., Pavliňák D., Ondreáš F., Jašek O., Štaffová M., Lepcio P. (2023). Matching Low Viscosity with Enhanced Conductivity in Vat Photopolymerization 3D Printing: Disparity in the Electric and Rheological Percolation Thresholds of Carbon-Based Nanofillers Is Controlled by the Matrix Type and Filler Dispersion. ACS Omega.

[203] Nashrullah F.M., Suryanto H., Aminnudin A., Bintara R.D., Maulana J. (2024). Analysis of the Addition of Nanographite on the Characteristics of Polylactic Acid Filaments Produced by Extrusion Process. BIO Web Conf..

[204] Rahman M.M., Khan K.H., Parvez M.M.H., Irizarry N., Uddin M.N. (2025). Polymer Nanocomposites with Optimized Nanoparticle Dispersion and Enhanced Functionalities for Industrial Applications. Processes.

[205] Luzzi E., de Salzano de Luna M., Caputo D., Filippone G., Aprea P. (2025). Printability Metrics in Direct Ink Writing: Critical Review of the Literature and Novel Perspective Based on Dimensional Analysis. Adv. Mater. Technol..

[206] Huangfu B., Liu Y., Liu X., Wu X., Bai H. (2024). Anisotropy of Additively Manufactured Metallic Materials. Materials.

[207] Frieden Templeton W., Hinnebusch S., Strayer S.T., To A.C., Pistorius P.C., Narra S.P. (2024). A mechanistic explanation of shrinkage porosity in laser powder bed fusion additive manufacturing. Acta Mater..

[208] Dai S., Zhu K., Wang S., Deng Z. (2025). Additively manufactured materials: A critical review on their anisotropic mechanical properties and modeling methods. J. Manuf. Process..

[209] Thanumoorthy R.S., Chaurasia J.K., Anil Kumar V., Pradeep P.I., Balan A.S.S., Rajasekaran B., Sahu A., Bontha S. (2024). Effect of Build Orientation on Anisotropy in Tensile Behavior of Laser Powder Bed Fusion Fabricated SS316L. J. Mater. Eng. Perform..

[210] Qiu H., Cui W., Xu T., Liu F., Chen Y., Yang K., Wang C., Liu Y., Wang Q. (2026). Decoupling Strengthening Mechanisms and Anisotropy in Laser Powder Bed Fusion Processed Al_0.3_CoCrFeNiMn High-Entropy Alloy. J. Mater. Eng. Perform..

[211] Liu K., Chen W., Shan F., Wang H., Li K. (2026). Printing-Path-Dominated Anisotropy in FDM-PEEK: Modulation by Build Orientation for Tensile and Shear Performance. Polymers.

[212] Cheadle A.M.G., Maier E., Palin W.M., Tomson P.L., Poologasundarampillai G., Hadis M.A. (2025). The impact of modifying 3D printing parameters on mechanical strength and physical properties in vat photopolymerisation. Sci. Rep..

[213] Shanmugasundaram S.A., Razmi J., Mian M.J., Ladani L. (2020). Mechanical anisotropy and surface roughness in additively manufactured parts fabricated by stereolithography (SLA) using statistical analysis. Materials.

[214] Naik D.L., Kiran R. (2018). On anisotropy, strain rate and size effects in vat photopolymerization based specimens. Addit. Manuf..

[215] Valizadeh I., Tayyarian T., Weeger O. (2023). Influence of process parameters on geometric and elasto-visco-plastic material properties in vat photopolymerization. Addit. Manuf..

[216] Tu J., Kashcooli Y., Alvarez N.J., Palmese G.R. (2022). A practical framework for predicting conversion profiles in vat photopolymerizations. Addit. Manuf..

[217] Tilve-Martinez D., Neri W., Vukadinovic N., Berton B., Pénicaud A., Yuan J., Poulin P. (2024). Electrical anisotropy and its mitigation in conductive polymers printed by vat photopolymerization. Addit. Manuf..

[218] Torres-Alvarez D., Celis-Guzman A., Aguirre-Soto A. (2025). Resin-dependent mechanical anisotropy in laser vat photopolymerization correlates to the initial rate of polymerization and critical energy. Addit. Manuf. Lett..

[219] Montgomery S.M., Yue L., Song Y., Nomura T., Sun X., Tanaka M., Qi H.J. (2023). Locally patterned anisotropy using grayscale vat photopolymerization. Addit. Manuf..

[220] Bonacini A., Saccani E., Sciancalepore C., Milanese D., Drago G., Pedrini A., Pinalli R., Nicolaÿ R., Dalcanale E. (2025). Boronate Esters Dynamic Networks for the Reduction of Mechanical Anisotropy in Vat 3D Printed Manufacts. ACS Appl. Polym. Mater..

[221] Greiner S., Schlicht S., Drummer D. (2025). New Process Strategies for Laser Powder Bed Fusion of Polymers. Progress in Powder Based Additive Manufacturing.

[222] Calignano F., Giuffrida F., Galati M. (2021). Effect of the build orientation on the mechanical performance of polymeric parts produced by multi jet fusion and selective laser sintering. J. Manuf. Process..

[223] Lee K.P.M., Pandelidi C., Kajtaz M. (2020). Build orientation effects on mechanical properties and porosity of polyamide-11 fabricated via multi jet fusion. Addit. Manuf..

[224] Kim S.I., Hart A.J. (2022). A spiral laser scanning routine for powder bed fusion inspired by natural predator-prey behaviour. Virtual Phys. Prototyp..

[225] Abbott C.S., Sperry M., Crane N.B. (2021). Relationships between porosity and mechanical properties of polyamide 12 parts produced using the laser sintering and multi-jet fusion powder bed fusion processes. J. Manuf. Process..

[226] Lavery N.P., Cherry J., Mehmood S., Davies H., Girling B., Sackett E., Brown S.G.R., Sienz J. (2017). Effects of hot isostatic pressing on the elastic modulus and tensile properties of 316L parts made by powder bed laser fusion. Mater. Sci. Eng. A.

[227] Shaffer S., Yang K., Vargas J., di Prima M.A., Voit W. (2014). On reducing anisotropy in 3D printed polymers via ionizing radiation. Polymer.

[228] Debreceni A., Buri Z., Bodzás S. (2025). Linking Process Parameters, Structure, and Properties in Material Extrusion Additive Manufacturing of Polymers and Composites: A Review. J. Manuf. Mater. Process..

[229] Uddin Z., Butt M.M., Kvvssn V., Salamci M.U., Kizil H. (2024). Understanding the Effects of Manufacturing Attributes on Damage Tolerance of Additively Manufactured Parts and Exploring Synergy Among Process-Structure-Properties. A Comprehensive Review. Eng. Rep..

[230] al Aridi R., DiNova V., Zhang T., Karna S., Yuan L., Krentz T., Hitchcock D., Gross A.J. (2024). Characterization of defects in additively manufactured materials from mechanical properties. Mater. Sci. Eng. A.

[231] Sanaei N., Fatemi A. (2021). Defects in additive manufactured metals and their effect on fatigue performance: A state-of-the-art review. Prog. Mater. Sci..

[232] Huang Y., Fleming T.G., Clark S.J., Marussi S., Fezzaa K., Thiyagalingam J., Leung C.L.A., Lee P.D. (2022). Keyhole fluctuation and pore formation mechanisms during laser powder bed fusion additive manufacturing. Nat. Commun..

[233] Yu T., Zhao J. (2023). Quantifying the mechanisms of keyhole pore evolutions and the role of metal-vapor condensation in laser powder bed fusion. Addit. Manuf..

[234] Avegnon K.L.M., Menendez A., Liu J., Mittal Y.G., Karunakaran K.P., Sealy M.P. (2023). Patterned keyhole porosity formation in laser powder bed fusion caused by local disturbances in the shielding gas flow. Manuf. Lett..

[235] Zhai Z., Wang S., Yang G., Guo A., Qu P., Song Y., Shao S., Zang J. (2025). Novel sandwich structures with double-row and crossed pyramidal lattice cores: Design, fabrication and bending behavior. Eng. Fail. Anal..

[236] Zhang D., Ma J., Liu L., Zhu Y., Guo A., Qu P., Guo S., Song Z., Song Y., Wang S. (2026). Mechanical Performance of Novel 3D-Printed Symmetric Corrugated Hierarchical Honeycombs. Polymers.

[237] Issametova M., Martyushev N.V., Zhastalap A., Sabirova L.B., Assemgul U., Tursynbayeva A., Abilezova G. (2024). Determination of Residual Stresses in 3D-Printed Polymer Parts. Polymers.

[238] Compton B.G., Post B.K., Duty C.E., Love L., Kunc V. (2017). Thermal analysis of additive manufacturing of large-scale thermoplastic polymer composites. Addit. Manuf..

[239] Barocio E., Brenken B., Favaloro A., Pipes R.B. (2022). Interlayer fusion bonding of semi-crystalline polymer composites in extrusion deposition additive manufacturing. Compos. Sci. Technol..

[240] Laleh M., Sadeghi E., Revilla R.I., Chao Q., Haghdadi N., Hughes A.E., Xu W., de Graeve I., Qian M., Gibson I. (2023). Heat treatment for metal additive manufacturing. Prog. Mater. Sci..

[241] Zhang G., Li N., Gao J., Xiong H., Yu H., Yuan H. (2022). Wire-fed electron beam directed energy deposition of Ti–6Al–2Zr–1Mo–1V alloy and the effect of annealing on the microstructure, texture, and anisotropy of tensile properties. Addit. Manuf..

[242] Gokcekaya O., Ishimoto T., Hibino S., Yasutomi J., Narushima T., Nakano T. (2021). Unique crystallographic texture formation in Inconel 718 by laser powder bed fusion and its effect on mechanical anisotropy. Acta Mater..

[243] Bera T., Mohanty S. (2024). A Review on Residual Stress in Metal Additive Manufacturing. 3D Print. Addit. Manuf..

[244] du Plessis A., Macdonald E. (2020). Hot isostatic pressing in metal additive manufacturing: X-ray tomography reveals details of pore closure. Addit. Manuf..

[245] Nudelis N., Mayr P. (2023). Pore tracing in additive manufactured and hot isostatic pressed components. J. Mater. Sci..

[246] Kaletsch A., Sondermann M., Mirz M., Radtke F., Broeckmann C. (2023). Influence of PBF-LB Process Atmosphere on the Fatigue Strength of Hot Isostatically Post-Densified Duplex Steel Parts Produced via the Shell Core Approach. Materials.

[247] Seok W., Jeon E., Kim Y. (2023). Effects of Annealing for Strength Enhancement of FDM 3D-Printed ABS Reinforced with Recycled Carbon Fiber. Polymers.

[248] Tejedor J., Cevallos P.D., Coro E.S., Pontón P.I., Guamán M., Guerrero V.H. (2025). Effects of annealing on the mechanical, thermal, and physical properties of 3D-printed PLA aged in salt water. Mech. Adv. Mater. Struct..

[249] Battistelli C., Seriani S., Lughi V., Slejko E.A. (2024). Optimizing 3D-Printing Parameters for Enhanced Mechanical Properties in Liquid Crystalline Polymer Components. Polym. Adv. Technol..

[250] Bouchareb S., Doufnoune R. (2025). Effect of UV post-curing on the mechanical properties of photopolymer resin in stereo-lithographic 3D printing. Mater. Lett..

[251] Büyükpolat M., Çal İ.K., Eryılmaz B., Ersöz B., Aydın N., Karaoğlanoğlu S. (2025). Mechanical and optical effects of post-curing time and device type in two 3D-printed resin systems. BMC Oral Health.

[252] Ahmad K.H., Mohamad Z., Khan Z.I. (2024). Influence of Graphene Nanoplatelets and Post-Curing Conditions on the Mechanical and Viscoelastic Properties of Stereolithography 3D-Printed Nanocomposites. Polymers.

[253] al Noman A., Kumar B.K., Dickens T. (2023). Field assisted additive manufacturing for polymers and metals: Materials and methods. Virtual Phys. Prototyp..

[254] Diaz Armas N., Bhandari G., Kodra S., Zhang J., Kazmer D., Mead J. (2025). Additive Manufacturing of Thermoset Elastomer–Thermoplastic Composites Using Dual-Extrusion Printing. Polymers.

[255] Goh G.D., Wong K.K., Tan N., Seet H.L., Nai M.L.S. (2024). Large-format additive manufacturing of polymers: A review of fabrication processes, materials, and design. Virtual Phys. Prototyp..

[256] Omer M.A.E., Shaban I.A., Mourad A.H., Hegab H. (2025). Advances in interlayer bonding in fused deposition modelling: A comprehensive review. Virtual Phys. Prototyp..

[257] Guessasma S., Nouri H., Belhabib S. (2022). Digital Image Correlation and Finite Element Computation to Reveal Mechanical Anisotropy in 3D Printing of Polymers. Materials.

[258] Jaganathan S., Kandasamy R., Venkatachalam R., Gunalan M., Dhairiyasamy R. (2024). Advances in Optimizing Mechanical Performance of 3D-Printed Polymer Composites: A Microstructural and Processing Enhancements Review. Adv. Polym. Technol..

[259] Yao J., Duongthipthewa A., Xu X., Liu M., Xiong Y., Zhou L. (2024). Interlayer bonding improvement of PEEK and CF-PEEK composites with laser-assisted fused deposition modeling. Compos. Commun..

[260] Andreu A., Kim S., Dittus J., Friedmann M., Fleischer J., Yoon Y.J. (2022). Hybrid material extrusion 3D printing to strengthen interlayer adhesion through hot rolling. Addit. Manuf..

[261] Gupta V., Bankapalli N.K., Saxena P., Bajpai A., Ruan D. (2025). Additive Manufacturing of Fiber-Reinforced Polymer Matrix Composites through Material Extrusion: A Comprehensive Review on Filament Fabrication, Printing, Testing Methods, Applications, and Challenges. Adv. Eng. Mater..

[262] Zhang J., Cao Q., Lu W.F. (2022). A review on design and removal of support structures in metal additive manufacturing. Mater. Today Proc..

[263] Jankowiak M., Niemann C., Weber J.U., Kelbassa I. (2025). Influencing factors of removability of support structures using high-frequency vibration. Prog. Addit. Manuf..

[264] Venturi F., Taylor R. (2023). Additive Manufacturing in the Context of Repeatability and Reliability. J. Mater. Eng. Perform..

[265] Zuo Z., de Corte W., Huang Y., Chen X., Zhang Y., Li J., Zhang L., Xiao J., Yuan Y., Zhang K. (2024). Strategies towards large-scale 3D printing without size constraints. Virtual Phys. Prototyp..

[266] Paxton N.C., Zhao J., Sauret E. (2024). Polymer 3D printing in perspective: Assessing challenges and opportunities in industrial translation against the metal benchmark. Int. J. Adv. Manuf. Technol..

[267] Ulkir O. (2023). Energy-Consumption-Based Life Cycle Assessment of Additive-Manufactured Product with Different Types of Materials. Polymers.

[268] al Rashid A., Koç M. (2023). Additive manufacturing for sustainability and circular economy: Needs, challenges, and opportunities for 3D printing of recycled polymeric waste. Mater. Today Sustain..

[269] Sharma A., Kumar M., Sharma A. (2025). Sustainable additive manufacturing: Challenges and opportunities of recycling plastic waste for 3D printing filaments. Sadhana Acad. Proc. Eng. Sci..

[270] Gomes P.C., Piñeiro O.G., Alves A.C., Carneiro O.S. (2022). On the Reuse of SLS Polyamide 12 Powder. Materials.

[271] Pham D.T., Dotchev K.D., Yusoff W.A.Y. (2008). Deterioration of polyamide powder properties in the laser sintering process. Proc. Inst. Mech. Eng. Part C J. Mech. Eng. Sci..

[272] Yang F., Zobeiry N., Mamidala R., Chen X. (2023). A review of aging, degradation, and reusability of PA12 powders in selective laser sintering additive manufacturing. Mater. Today Commun..

[273] Sola A., Trinchi A. (2023). Recycling as a Key Enabler for Sustainable Additive Manufacturing of Polymer Composites: A Critical Perspective on Fused Filament Fabrication. Polymers.

[274] Jarach N., Dodiuk H., Kenig S., Magdassi S. (2024). Fully Recyclable Cured Polymers for Sustainable 3D Printing. Adv. Mater..

[275] Su J., Ng W.L., An J., Yeong W.Y., Chua C.K., Sing S.L. (2024). Achieving sustainability by additive manufacturing: A state-of-the-art review and perspectives. Virtual Phys. Prototyp..

[276] Yan T., Balzer A.H., Herbert K.M., Epps T.H., Korley L.S.T.J. (2023). Circularity in polymers: Addressing performance and sustainability challenges using dynamic covalent chemistries. Chem. Sci..

[277] Sun M., Felsenthal L.M., Kim S., Choi E.Y., Reed L.J., Elling B.R., Dichtel W.R. (2026). Covalent Adaptable Networks: Reprocessable Cross-Linked Polymers. Chem. Rev..

[278] Ecker J., Liska R., Stampfl J. (2024). Design for disassembly: Using a multi-material approach in 3D printing for easier recycling strategies. Addit. Manuf..

[279] Frascio M., Morchio S., Musiari F., Muhammad Usman K., Dittamo F., Minuto M., Avalle M. (2025). Investigating Multi-Material Additive Manufacturing for Disassembly and Reparability of Adhesive Joints by Precision Heating. Adhesives.

[280] Saitta L., Dattilo S., Tosto C., Giglio V., Riccobene P.M., Blanco I., Latteri A., Cicala G. (2026). Epoxy Latent Systems for Novel Hybrid 3D Printed Metal/CFs Reinforced Composite Joint Disassembly via Chemical Recycling. J. Polym. Environ..

[281] Kim S., Moon S.K. (2020). A part consolidation design method for additive manufacturing based on product disassembly complexity. Appl. Sci..

[282] Nguyen T.D., Nguyen M.T.N., Lee J.S. (2025). Dynamic Covalent Bonds in 3D-Printed Polymers: Strategies, Principles, and Applications. Appl. Sci..

[283] Das A., Sarmah A., Luster L.E., Green M.J., Bortner M.J. (2025). Additive manufacturing of vitrimers: Interplay between polymer physics and processing approaches. Chem. Eng. J..

[284] Karatrantos A.V., Couture O., Hesse C., Schmidt D.F. (2024). Molecular Simulation of Covalent Adaptable Networks and Vitrimers: A Review. Polymers.

[285] Krishnakumar B., Sanka R.V.S.P., Binder W.H., Parthasarthy V., Rana S., Karak N. (2020). Vitrimers: Associative dynamic covalent adaptive networks in thermoset polymers. Chem. Eng. J..

[286] Shi X., Zhuang D. (2026). Thermosets Based on Covalent Bond Exchange: Mechanisms, Properties, and Reprocessing. Polymers.

[287] Maes S., Badi N., Winne J.M., du Prez F.E. (2025). Taking dynamic covalent chemistry out of the lab and into reprocessable industrial thermosets. Nat. Rev. Chem..

[288] Beltrán F.R., Infante C., de la Orden M.U., Martínez Urreaga J. (2019). Mechanical recycling of poly(lactic acid): Evaluation of a chain extender and a peroxide as additives for upgrading the recycled plastic. J. Clean. Prod..

[289] Benvenuta-Tapia J.J., Champagne P., Tenorio-López J.A., Vivaldo-Lima E., Guerrero-Santos R. (2021). Improving recycled poly(Lactic acid) biopolymer properties by chain extension using block copolymers synthesized by nitroxide-mediated polymerization (nmp). Polymers.

[290] Ozmen S.C., Ozkoc G., Serhatli E. (2019). Thermal, mechanical and physical properties of chain extended recycled polyamide 6 via reactive extrusion: Effect of chain extender types. Polym. Degrad. Stab..

[291] Albertini E., Dalle Vacche S., Vitale A. (2026). Bio-based vitrimers: Chemistry, performance and applications. RSC Appl. Polym..

[292] Ponis S., Aretoulaki E., Maroutas T.N., Plakas G., Dimogiorgi K. (2021). A systematic literature review on additive manufacturing in the context of circular economy. Sustainability.

[293] Mad Yusoh S.S., Abd Wahab D., Habeeb H.A., Azman A.H. (2021). Intelligent systems for additive manufacturing-based repair in remanufacturing: A systematic review of its potential. PeerJ Comput. Sci..

[294] Nipu S.M.A., Tang T., Joralmon D., Liu T., Li R., Yoo M., Li X. (2025). Advances and perspectives in multi-material additive manufacturing of heterogenous metal-polymer components. npj Adv. Manuf..

[295] Belei C., Effertz P.S., Meier B., Amancio-Filho S.T. (2023). Additive manufacturing of metal-polymer hybrid parts: The influence of as-printed LPBF surface roughness on the joint strength. Front. Mater..

[296] Vakharia V.S., Leonard H., Singh M., Halbig M.C. (2023). Multi-Material Additive Manufacturing of High Temperature Polyetherimide (PEI)–Based Polymer Systems for Lightweight Aerospace Applications. Polymers.

[297] Zhang H., Wang P., Chen Z., Chen X., Jiang M., Yang J., Li J. (2024). Functional metallic circuitries created by laser-activated selective electroless plating for 3D customized electronics. Mater. Des..

[298] Akin S., Nath C., Jun M.B.G. (2023). Selective Surface Metallization of 3D-Printed Polymers by Cold-Spray-Assisted Electroless Deposition. ACS Appl. Electron. Mater..

[299] Li J., Liang Z., Li T., Wan Q., Huang X., Kan Q. (2025). A comprehensive review on 4D printing and applications of thermo-induced shape memory polymers. Int. J. Smart Nano Mater..

[300] Zhang Y., Huang L., Song H., Ni C., Wu J., Zhao Q., Xie T. (2019). 4D Printing of a Digital Shape Memory Polymer with Tunable High Performance. ACS Appl. Mater. Interfaces.

[301] Siddique M.F., Omar F.K., Al-Marzouqi A.H. (2026). Design and Application of Stimuli-Responsive Hydrogels for 4D Printing: A Review of Adaptive Materials in Engineering. Gels.

[302] Nakamura K., di Caprio N., Burdick J.A. (2024). Engineered Shape-Morphing Transitions in Hydrogels Through Suspension Bath Printing of Temperature-Responsive Granular Hydrogel Inks. Adv. Mater..

[303] Liu G., Wu J., Yang Y., Luo J., Xie X. (2026). 4D Printing in Regenerative Medicine: Bio-Inspired Applications for Dynamic Tissue Repair. J. Funct. Biomater..

[304] Shen C., Shen A. (2025). 4D printing: Innovative solutions and technological advances in orthopedic repair and reconstruction, personalized treatment and drug delivery. BioMed. Eng. Online.

[305] Kim H., Kim K.-H., Jeong J., Jeon H., Jung I.D. (2025). Advancing intelligent additive manufacturing: Machine learning approaches for process optimization and quality control. Int. J. AI Mater. Des..

[306] Ng W.L., Goh G.L., Goh G.D., Ten J.S.J., Yeong W.Y. (2024). Progress and Opportunities for Machine Learning in Materials and Processes of Additive Manufacturing. Adv. Mater..

[307] Karuppusamy M., Thirumalaisamy R., Palanisamy S., Nagamalai S., el Sayed Massoud E., Ayrilmis N. (2025). A review of machine learning applications in polymer composites: Advancements, challenges, and future prospects. J. Mater. Chem. A.

[308] Shivajirao P.S., Shailaja P., Snehal B. Real-Time Defect Detection in 3D Printing Using Deep Learning. Proceedings of the 2024 IEEE 9th International Conference for Convergence in Technology, I2CT 2024.

[309] Farhan Khan M., Alam A., Ateeb Siddiqui M., Saad Alam M., Rafat Y., Salik N., Al-Saidan I. (2020). Real-time defect detection in 3D printing using machine learning. Mater. Today Proc..

[310] Hu W.J., Chen C., Su S., Zhang J., Zhu A. (2024). Real-time defect detection for FFF 3D printing using lightweight model deployment. Int. J. Adv. Manuf. Technol..

[311] Long T., Pang Q., Deng Y., Pang X., Zhang Y., Yang R., Zhou C. (2025). Recent Progress of Artificial Intelligence Application in Polymer Materials. Polymers.

[312] Zhang Z., Wang Y., Wang W. (2025). Machine Learning in Gel-Based Additive Manufacturing: From Material Design to Process Optimization. Gels.

[313] Koltsakidis S., Tzimtzimis E.K., Tzetzis D. (2026). Machine Learning for Predicting Mechanical Properties of 3D-Printed Polymers from Process Parameters: A Review. Polymers.

[314] Zhang X., Chu D., Zhao X., Gao C., Lu L., He Y., Bai W. (2024). Machine learning-driven 3D printing: A review. Appl. Mater. Today.

[315] Guo H., Li S., Li S. (2026). Applications of Machine Learning in Polymer Materials: Property Prediction, Material Design, and Systematic Processes. Comput. Mater. Contin..

[316] Ramprasad M., Kim C. (2020). Assessing and Improving Machine Learning Model Predictions of Polymer Glass Transition Temperatures. J. Emerg. Investig..

[317] Pai S.M., Shah K.A., Sunder S., Albuquerque R.Q., Brütting C., Ruckdäschel H. (2025). Machine learning applied to the design and optimization of polymeric materials: A review. Next Mater..

[318] Zhao Y., Mulder R.J., Houshyar S., Le T.C. (2023). A review on the application of molecular descriptors and machine learning in polymer design. Polym. Chem..

[319] Fu H., Zobeiry N. (2026). Data-Driven Machine Learning Meta-Analysis of Process–Property Relationships in Polymer Additive Manufacturing: A Case Study on FFF-Printed PEEK. J. Manuf. Process..

[320] Sridhar S., Venkatesh K., Revathy G., Venkatesan M., Venkatraman R. (2025). Adaptive fabrication of material extrusion-AM process using machine learning algorithms for print process optimization. J. Intell. Manuf..

[321] Asadollahi-Yazdi E., Gardan J., Lafon P. (2018). Multi-Objective Optimization of Additive Manufacturing Process. IFAC PapersOnLine.

[322] Tura A.D., Mamo H.B. (2022). Characterization and parametric optimization of additive manufacturing process for enhancing mechanical properties. Heliyon.

[323] Deepa N., Mary S.A.S.A., Devi R.M., Husain S.S., Uddaraju S., Muniyandy E. (2026). Reinforcement learning-based closed-loop control system for adaptive process parameter optimisation in 3D printing. Nondestruct. Test. Eval..

[324] Suresh T., Deepapriya B.S., Suganya S., Murugaveni S., Waris S.F., Muniyandy E. (2026). Closed-loop reinforcement learning control of AM process parameters using Digital Twin feedback for defect mitigation and print correction. Nondestruct. Test. Eval..

[325] Shevchik S.A., Masinelli G., Kenel C., Leinenbach C., Wasmer K. (2019). Deep learning for in situ and real-time quality monitoring in additive manufacturing using acoustic emission. IEEE Trans. Ind. Inform..

[326] Oster S., Breese P.P., Ulbricht A., Mohr G., Altenburg S.J. (2024). A deep learning framework for defect prediction based on thermographic in-situ monitoring in laser powder bed fusion. J. Intell. Manuf..

[327] Feng S., Chen Z., Bircher B., Ji Z., Nyborg L., Bigot S. (2022). Predicting laser powder bed fusion defects through in-process monitoring data and machine learning. Mater. Des..

[328] Magolon M., Boer J., Elbestawi M. (2026). Defect Monitoring of Complex Geometries Through Machine Learning in LPBF Metal Additive Manufacturing. J. Manuf. Mater. Process..

[329] Abiria I., Wang C., Zhang Q., Liu C., Jin X. (2025). High-cycle and very-high-cycle fatigue life prediction in additive manufacturing using hybrid physics-informed neural networks. Eng. Fract. Mech..

[330] Salvati E., Tognan A., Laurenti L., Pelegatti M., de Bona F. (2022). A defect-based physics-informed machine learning framework for fatigue finite life prediction in additive manufacturing. Mater. Des..

[331] Wang H., Li B., Lei L., Xuan F. (2024). Uncertainty-aware fatigue-life prediction of additively manufactured Hastelloy X superalloy using a physics-informed probabilistic neural network. Reliab. Eng. Syst. Saf..

[332] Mehdipour S., Habibzadeh A., Esmi N., Heravi P., Mirsaraee M., Shahbahrami A., Javanmardi Z. (2026). Integrating digital twins, data management and artificial intelligence for predictive maintenance in aviation: A comprehensive review. Digit. Twins Appl..

[333] Hartwell A., Montana F., Jacobs W., Kadirkamanathan V., Ameri N., Mills A.R. (2024). Distributed digital twins for health monitoring: Resource constrained aero-engine fleet management. Aeronaut. J..

[334] Jin L., Zhai X., Wang K., Zhang K., Wu D., Nazir A., Jiang J., Liao W.H. (2024). Big data, machine learning, and digital twin assisted additive manufacturing: A review. Mater. Des..

[335] Khan I., al Rashid A., Koç M. (2025). Integration of machine learning and digital twin in additive manufacturing of polymeric-based materials and products. Prog. Addit. Manuf..

[336] Ahsan M.M., Liu Y., Raman S., Siddique Z. (2025). Digital Twins in Additive Manufacturing: A systematic review. Internet Things.

[337] Cooley I., Wang W., Kozyrev V., Wildman R.D., Johnston B.F., Croft A.K. (2025). Predictive Approaches for 3D-Printing: Methods and Approaches for Polymeric Materials. Wiley Interdiscip. Rev. Comput. Mol. Sci..

[338] Yue T., He J., Li Y. (2025). Machine-Learning-Assisted Molecular Design of Innovative Polymers. Acc. Mater. Res..

[339] Li X. (2025). Multiscale computational modeling of 3D printed continuous Fiber reinforced polymer composites. Sci. Rep..

[340] ben Amor S., Elloumi N., Eltaief A., Louhichi B., Alrasheedi N.H., Seibi A. (2024). Digital Twin Implementation in Additive Manufacturing: A Comprehensive Review. Processes.

[341] Fu Y., Downey A.R.J., Yuan L., Huang H.T., Ogunniyi E.A. (2025). Simulation-in-the-loop additive manufacturing for real-time structural validation and digital twin development. Addit. Manuf..

[342] Zaidi A.A., Asif M., Aljabri A., Khan S.Z. (2026). Intelligent Composite 3D Printing: The Role of Artificial Intelligence, Machine Learning, and In-Situ Monitoring in Next-Generation Additive Manufacturing. Front. Mech. Eng..

